# Vincular Project: study protocol for a randomized controlled trial with changes in 24-h movement behaviors targeting the reduce of depressive symptoms in adults

**DOI:** 10.1186/s13063-026-09771-8

**Published:** 2026-05-15

**Authors:** Cecília Bertuol, Deborah Kazimoto Alves, Giovani Firpo Del Duca

**Affiliations:** Federal University of Santa Catarina, Sports Center, Campus Universitário Reitor João David Ferreira Lima, Centro de Desportos, Florianópolis, SC 88040-900 Brazil

**Keywords:** Adults, 24-h movement behaviors, Depression, Clinical trial, Basic psychological needs, Protocol

## Abstract

**Background:**

A better understanding of how behavior change interventions work to reduce depressive symptoms, as well as their effects and potential mediators, is needed. This paper described the methods of the Vincular Project, an intervention based on Self-Determination Theory with changes in 24-h movement behaviors (physical activity, sedentary behavior, and sleep) to reduce depressive symptoms in Brazilian adults.

**Methods:**

This is a randomized, controlled, blinded clinical trial with adults aged 20 to 59 years, of both sexes, with depressive symptoms. The sample was intentional and non-probabilistic and participants were allocated to a control group and an intervention group. The intervention was carried out twice a week, for 16 weeks, and consisted of face-to-face and online meetings. The activities aimed to make participants more aware of their living and health conditions, exploring 24-h movement behaviors, especially physical activity, and their relationships with depressive symptoms. The contents were different in each meeting and the activities were organized into four blocks, the first being contextualization, the second with general and specific guidelines on 24-h movement behaviors, the third with opportunities to practice physical activity in different contexts and situations, and the fourth with strategies for maintaining healthy behaviors in the short and long term. Baseline (January 2023), post-intervention (May 2023), and maintenance (November 2023) evaluations included depressive symptoms as primary outcome, and 24-h movement behaviors and basic psychological needs as secondary outcomes. The variables of autonomy, competence, and relatedness were also tested as mediators related to the effectiveness of the intervention on depressive symptoms. Depressive symptoms were measured by the Patient Health Questionnaire-9 (PHQ-9), 24-h movement behaviors by self-report and accelerometry, and basic psychological needs by the translated and adapted version of the Basic Psychological Needs in Exercise Scale (BPNES). To verify the effect of the intervention, per-protocol and intention-to-treat analyses will be conducted using generalized estimating equations (GEE). Structural equation models (SEMs) will be employed in the mediation analysis.

**Discussion:**

The results of this trial will involve valuable information about the effect of a behavioral intervention to reduce depressive symptoms carried out in a middle-income country.

**Trial registration:**

Brazilian Registry of Clinical Trials (ReBEC): RBR-7466htj. Approved 14 April 2023, https://ensaiosclinicos.gov.br/rg/RBR-7466htj

**Supplementary Information:**

The online version contains supplementary material available at 10.1186/s13063-026-09771-8.

## Introduction

Currently, depression represents one of the biggest causes of disability, being the disorder with the greatest impact on the global burden of disease [1, 2] and being associated with a higher rate of premature mortality [3, 4]. This chronic non-communicable disease affects more than 300 million people of all ages, which is equivalent to approximately 4.4% of the population globally [1, 2], generating a high cost to the global economy [5]. Considering that depression is often underdiagnosed and undertreated [5, 6], there is a need for specific interventions that can contribute to a more stable remission, prolonging the time between depressive episodes and reducing relapse rates, as well as monitoring their indicators to better control this scenario.

Although psychotherapy and pharmacotherapy are the most recognized treatments for depressive symptoms and depression [7, 8], recent evidence highlights that regular physical activity can be considered an additional, effective, and independent option [9–12]. This is because aspects related to lifestyle—which are modifiable—are strongly associated with depressive episodes [13–17]. Along with physical activity, sedentary behavior and sleep, known as 24-h movement behaviors, have been gaining notoriety in the context of mental illnesses, mainly due to the fact that these behaviors contribute simultaneously to the prevention and treatment of depression and depressive symptoms, for example [13, 14, 16].

Systematic reviews and meta-analysis studies confirm the hypothesis that physical inactivity [18, 19], high sedentary behavior [20, 21], short and long sleep duration [22], and sleeping problems, such as insomnia [23, 24], are associated with greater chances of developing depression. However, the reverse direction of these associations may also occur, suggesting that individuals with depressive symptoms are more likely to be physically inactive or not meet physical activity recommendations [13, 15, 16, 19], to present high levels of sedentary behavior [13, 16], and to develop sleep disorders [14, 17], in compared to those without a diagnosis of this disease. As for clinical trials investigating these behaviors and depressive episodes, it is possible to see positive results, with physical activity providing antidepressant effects on the disease [19] and sleep bringing improvements in general mental health, depression, and other mental health problems, such as anxiety, stress, and rumination [14]. For sedentary behavior, what generally occurs is the development of interventions in which physically active participants are exposed to a reduction in the physical activities they usually do for, approximately, 1 week with the purpose of examining the effect of these changes on symptoms of mood, stress, and depression [25–27].

Furthermore, little is known about the long-term effects of these interventions. In addition to the heterogeneous data on monitoring and maintenance [28], there is a lack of studies regarding the remission of depression symptoms and the continuity of healthy behaviors [29]. Although short-term results can be achieved for both depressive symptoms [9, 11, 12] and 24-h movement behaviors [14, 19, 25], it is crucial to emphasize the significance of investigating these variables in the long term, months after the interventions have concluded. This approach is essential for filling critical gaps in understanding the enduring impacts of these interventions. Despite the benefits of a healthy lifestyle, individuals with depressive symptoms experience barriers such as the inherent symptomatology of the disease, leading to a lack of motivation and loss of interest [2], common factors for discontinuation of health promotion efforts across various contexts.

In this sense, the implementation of resources that address multicomponent actions, related to the integrated management of 24-h movement behaviors, and that adopt adherence and maintenance strategies may be a more effective method compared to traditional trials, which involve segmented approaches to these behaviors, in an attempt to reduce symptoms of depression in the adult population. Moreover, considering the low adherence of this population to programs of this nature [30, 31] and given that these behaviors are influenced by several factors, such as sociodemographic, lifestyle, and health conditions [16], it is also necessary to understand the mechanisms that can trigger psychological aspects that favor behavior change processes.

For this purpose, Self-Determination Theory, for instance, contributes to a better understanding of the different types of motivation that lead individuals to either adopt or refrain from certain behaviors [32]. The more autonomous (or self-determined) an individual is, the more adaptive the health outcomes, favoring behavioral adoption and maintenance [32, 33]. Additionally, it is observed that more autonomous motivation is facilitated by the satisfaction of three basic psychological needs, including autonomy (feeling empowered and having the power of choice), competence (feeling capable and effective), and relatedness (feeling connected, close, and valued by others) [32, 34]. Whether an individual’s psychological needs are met or not depends mainly on the extent to which the environment and relationships support or frustrate these needs [34, 35]. Thus, when considering interventions aimed at behavior change with a focus on reducing depressive symptoms, it is evident that the satisfaction of basic psychological needs can be considered an important mediator in the relationship between the proposed programs and the investigated outcomes [36]. Nevertheless, there remains a challenge for healthcare professionals in facilitating the satisfaction of these needs more effectively, particularly concerning techniques and strategies [37], especially in mental health outcomes.

In light of the above, the objective of this paper is to provide a step-by-step description of the design of Vincular Project, a behavior change intervention based on Self-Determination Theory for the reduction of depressive symptoms in Brazilian adults. The intervention is grounded not only in the main outcomes (depressive symptoms and 24-h movement behaviors) but also in potential mediating factors (basic psychological needs) and health-related outcomes (e.g., lifestyle), as well as assessment of maintenance of long-term effects.

## Methods

### The Vincular Project aim and study design overview

Considering that the intervention proposal addressed aspects of both the body and mind, in addition to the constructs of the Self-Determination Theory, the project was named “Vincular” (the Portuguese word for “to link” or “to connect”), in the sense of intertwining, connecting, and incorporating all these elements. Additionally, the word ends in “lar” (which, when translated into English, means “home”), precisely representing the intended creation of a welcoming, safe, receptive environment free from judgment. The main objective was to promote different dynamics based on the Self-Determination Theory, with a focus on 24-h movement behaviors, especially physical activity, to reduce depression symptoms in adults from Florianópolis, a city located in the southern region of Brazil. Furthermore, as secondary objectives, the study aimed to investigate the effects of the program on indicators of 24-h movement behaviors (physical activity, sedentary behavior, and sleep) and on basic psychological needs (autonomy, competence, and relatedness), as well as verifying its role mediator in achieving primary results.

The recommendations of the SPIRIT (Standard Protocol Items: Recommendations for Interventional Trials) were followed to guide the construction of the present study [38]. The Vincular Project is a parallel-group, randomized controlled clinical trial with blinding of the primary, secondary outcomes, and other variables, following the design outlined by Hulley et al. [39]. It consists of two arms (a control group and an intervention group) and is conducted at a single center. This study was registered in the Brazilian Registry of Clinical Trials (ReBEC) under the identification code RBR-7466htj, and submitted to the Research Ethics Committee involving Human Subjects at the Federal University of Santa Catarina (CEPSH-UFSC), obtaining a favorable opinion (Opinion Number 5.622.082; CAAE 60378122.1.0000.0121). The informed consent form was made available online, accessible for download, and participants indicated their agreement or disagreement to participate in the research.

### Target population and sample

The target population of the present study consisted of adults aged 20 to 59 years, representing the adult age group as defined by the Ministry of Health and the World Health Organization [40, 41], of both sexes, and experiencing depressive symptoms. The sample was intentional and non-probabilistic, comprising adult individuals with depressive symptoms residing in Florianópolis, within the aforementioned age range, and expressing interest in participating in the study.

### Inclusion criteria

- Score ≥ 9 points on the Patient Health Questionnaire-9 (PHQ-9);
- Be aged between 20 and 59 years;
- Possess an electronic device with a camera and microphone, such as a computer, tablet, or cell phone;
- Have access to the internet;
- Be available for online and in-person meetings on specified days and times;
- Not be pregnant or in the postpartum period.

### Exclusion criteria

- Require specialized psychiatric treatment, excluding cases of depression (e.g., psychosis, schizophrenia) based on self-reported diagnosis;
- Exhibit suicidal risk, as indicated by selecting the response options “a week or more” or “almost every day” in the PHQ-9 suicidal risk question;
- Have illnesses or physical limitations that impede participation in the study.

### Sample size, randomization, and allocation concealment

The sample size was calculated using G*power 3.1.9.2 software, and was based on an ANOVA model for repeated measures with three time points and two groups. Parameters included an average effect size of 0.25, statistical power of 80%, and 5% significance level for two-tailed tests, reaching a value of 28 individuals. Anticipating a potential dropout rate of up to 50%, the number calculated was doubled, bringing the total sample size to 56 individuals. Eligible individuals were randomly assigned to either the control group (CG), instructed to maintain their usual activities, or the intervention group (IG), which actively participated in the program. To ensure numerical and participant characteristic balance between groups, randomization was stratified by sex, age, and PHQ-9 score, employing a 1:1 ratio. The randomization process was executed using the online platform randomizar.org by researchers not directly involved in the intervention. The allocation list was concealed from all study evaluators.

### Recruitment, screening, and selection

The sample recruitment process commenced after receiving approval from the CEPSH-UFSC. A comprehensive publicity strategy was implemented both online and in person. Initially, information was disseminated on the internet through social networks and email lists. Subsequently, posters were displayed at the Federal University of Santa Catarina, the University Hospital, and Basic Health Units, and letters were distributed to homes near the university. Additionally, the study was promoted through various channels, including a local program (TV UFSC), a television news segment featured in the “Jornal do Almoço,” a Brazilian television program aired by NSC-TV, an affiliate of Rede Globo, and a podcast called “Vem Cienciar.” Links to all these features were also made available online.

Those interested in participating in the Vincular Project could reach out through phone calls, WhatsApp messages, and emails. An initial registration process was facilitated via an online form-filling platform, allowing for the collection of identification information and a preliminary screening to verify compliance with eligibility criteria. Individuals who did not fit the requested profile were dismissed and those who met the pre-established criteria were invited to participate in a meeting for (a) guidance on the research objectives and procedures; and (b) completing additional questionnaires and receiving accelerometers. It is important to emphasize that the application of the informed consent form was administered prior to any data collection instrument. This form was made available online, enabling interested parties to review and express their agreement or refusal to participate in the research. Furthermore, at the conclusion of this session, the term, initialed and signed by the responsible researcher, was made available for download, as mentioned before.

It is noteworthy that psychoeducational materials on mental health were made available and referrals were provided to those who were not included in the research and who presented a degree of suicidal risk. During all phases of the research, other support materials were also furnished. These included the Brazilian Guide to Physical Activity, which presents comprehensive guidelines for engaging in physical activity and addressing sedentary behaviors in a didactic manner. Moreover, a compilation of tools for recognizing and treating depression, published by the Brazilian Medical Association in the Brazilian Journal of Psychiatry, was supplied. Informative manuals and booklets focusing on mental health, disseminated by the World Health Organization, the Pan-American Health Organization, the Ministry of Health, and the Brazilian Psychiatric Association, were also distributed. All individuals interested in volunteering for the research and who passed the screening of the eligibility criteria were welcomed in the same way, receiving all the necessary guidance and direction, based on their individual responses.

### Assessments

**Fig. 1:**
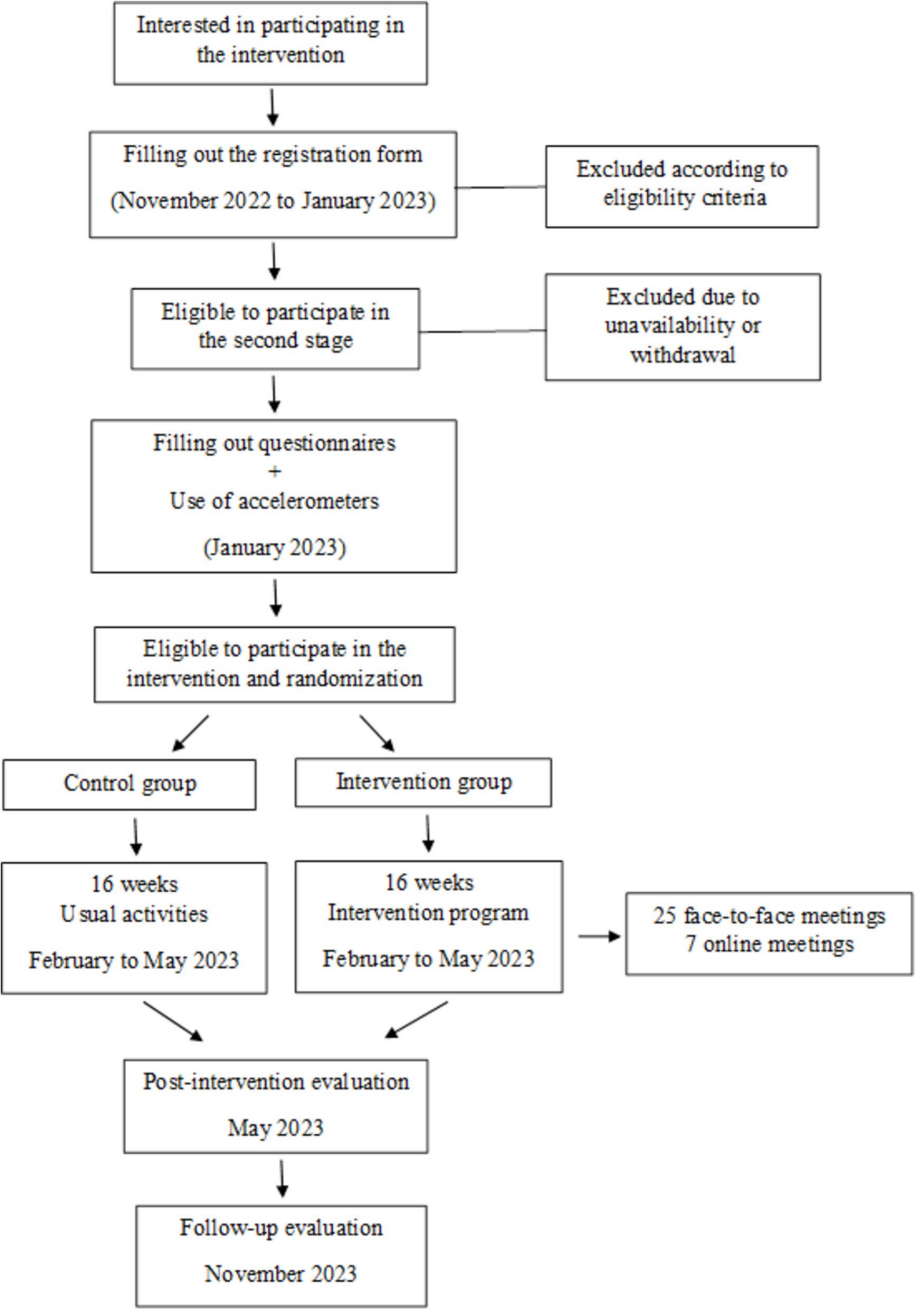
Study design flowchart. Source: own authorship (2023)

The assessment of the variables of interest was carried out at three moments: (a) baseline pre-intervention (January 2023); (b) post-intervention, immediately following the conclusion of the program (May 2023); and (c) post-intervention, 6 months after the end of the intervention (follow-up evaluation, conducted in November 2023). Questionnaires were employed to measure the variables, and participants were required to respond in the presence of a previously trained researcher. To facilitate this process, specific dates and times were scheduled based on participants’ availability. Furthermore, during these assessment periods, participants used accelerometers to objectively measure their 24-h movement behaviors. In Fig. 1, it is possible to observe a schematic diagram with the time schedule of enrolment, interventions, and assessments.

### Theoretical basis of the Vincular Project

Various program frameworks and behavior change theories have contributed to the development of the intervention. The Self-Determination Theory, formulated by Edward L. Deci and Richard M. Ryan in 1981, emphasizes the socio-contextual conditions that either facilitate or impede the natural processes of self-motivation and healthy psychological development [33]. This theory is grounded in an approach to motivation and human personality that employs traditional empirical methods by applying an organismic metatheory (explained below), which highlights the importance of the internal resources of human beings in order to enable personality development and behavioral self-regulation [33, 42]. One of the main objectives of the theory has been to offer a more specific focus on motivation, seeking to understand what type of stimulus is being manifested at a given moment or driving a particular behavior [33].

In this context, Self-Determination Theory focuses on differentiating between self-determined (also known as autonomous) and non-self-determined (or controlled) forms of motivation, which reflect the extent to which actions are fully endorsed by the individual [33]. Self-determined motivation pertains to reasons or motives for engaging in a behavior or achieving a specific goal that are self-endorsed. Individuals driven by autonomous motives perceive their actions as freely chosen and aligned with their personal beliefs, values, and goals [43]. Conversely, non-self-determined motivation encompasses reasons for action that are not self-endorsed. In this case, individuals who report controlled reasons for their actions perceive their behaviors as originating from external sources, being controlled by external circumstances or contingencies [44, 45]. There is a third form of regulation called amotivation, which consists of the lack of any motivational force to act. Individuals experiencing amotivation do not recognize any clear reason, motive, or intention behind their actions [32, 46]. It is evident, therefore, that motivation can be influenced and directed by numerous internal and external factors, each with varying effects on individuals’ behavior [47].

In order to detail the different forms of motivation and the contextual factors that facilitate or impede the internalization and integration of the regulation of these behaviors, Deci and Ryan [35] developed a subtheory within Self-Determination Theory, known as organismic integration theory. This theory delineates a continuum of self-determination wherein amotivation, or the absence of intent to act, resides at the left end. As previously mentioned, unmotivated individuals either abstain from action or engage without any underlying motivation, merely going through the motions. Moving along the continuum, there is the extrinsic motivation originating from external sources. This type of motivation involves performing an action to attain a separable outcome and lacks inherent satisfaction from the activity itself. Extrinsic motivation can be categorized based on four regulatory styles. External regulation encompasses less autonomous behaviors performed to meet an external demand or respond to a reward or threat. Introjected regulation reflects the acceptance of a regulation, albeit not entirely internalized, wherein behaviors are executed to alleviate guilt or anxiety or to boost one’s ego, maintaining a sense of value, such as pride. The third type of extrinsic motivation is identified regulation, which reflects a conscious appreciation of a goal or behavioral regulation, leading to the acceptance or acknowledgment of the action as personally important, beneficial, and useful. Finally, integrated regulation is characterized as the most autonomous form of extrinsic motivation, in which behaviors are assessed and recognized as significant and consistent with other values and needs. It is noteworthy that external and introjected regulations together constitute a composite of controlled (non-self-determined) motivation, while identified and integrated regulations have autonomous (self-determined) components. At the far right of the continuum, intrinsic motivation corresponds to a process characterized by personal choice, satisfaction, and pleasure.

Another assumption of Self-Determination Theory is that the level and quality of motivation experienced by individuals during the performance of an action are determined by the perceived value of that action in satisfying three fundamental psychological needs: autonomy, competence, and relatedness [32, 34]. Autonomy refers to actions that are self-endorsed, freely chosen by the individual, leading to a sense of ownership and personal responsibility. Competence corresponds to the perception of effectiveness in interactions with the environment, of mastering and expanding your capabilities and skills, making them challenging, until personal growth is achieved. The relatedness revolves around the desire for belonging, close emotional relationships, and the need to feel accepted and respected, fostering a sense of connection and mutual concern. It is evident that these basic psychological needs are innate and intricately linked to intrinsic motivation, self-regulation, and overall well-being. They explore the processes and conditions that facilitate healthy development and effective functioning of the individual [32]. Additionally, it is highlighted that these psychological needs are interdependent, suggesting that “the satisfaction of one need supports the satisfaction of the other two needs” [34].

Whether an individual’s basic psychological needs are satisfied or frustrated depends on the extent to which the subject’s environment and relationships support or harm these needs [34, 35]. Behaviors or messages from social agents (such as health professionals, family, friends, and colleagues), who operate within an individual’s social environment, or information obtained through various channels like magazines, newspapers, or social networks, can contribute to changes in psychological needs [36]. For example, if these behaviors, messages, or content support the satisfaction of psychological needs, individuals will presumably experience their actions as autonomously motivated, enabling the engagement and/or maintenance of health behaviors [34–36].

On the other hand, behaviors, messages, or content that do not support or impede the satisfaction of psychological needs may possibly harm autonomous motivation. This, in turn, can favor controlled (non-self-determined) forms of motivation or amotivation, potentially contributing to maladaptive results and behavioral disengagement [34–36]. Teixeira and collaborators [36] state that “guidance on the behaviors displayed by social agents, and specifying the content of messages, are potentially effective means to promote autonomous motivation and sustained behavior change.” They also emphasize the importance of understanding how these interventions influence the quality of motivation through the satisfaction of the three basic psychological needs. From this perspective, the satisfaction of basic psychological needs may be considered an important mediator of interventions based on the Self-Determination Theory on health outcomes, especially behavior change, reflecting both mental and physical aspects [36].

Ryan and Deci [33] offer additional examples of how basic psychological needs can influence the different regulations of extrinsic motivation. Since extrinsically motivated behaviors are not typically interesting, it becomes clear that many individuals engage in actions based on their connection to or desire for connection with people close to them. These individuals feel a sense of connection or relationship with others who request, model, or value these behaviors. This suggests that the relatedness, the need to belong, is a crucial element for internalization. Perceived competence also plays a role in the relative internalization of extrinsically motivated activities, as individuals tend to adopt activities or behaviors valued by social groups when they perceive themselves as capable and effective in relation to those activities. To complete the triangle of psychological needs, autonomy supports internalization and, in particular, is a critical component for the integration of regulation.

Demonstrations of positive results associated with Self-Determination Theory can be observed in different behaviors, contexts, and populations. A systematic review with the aim of investigating the relationships between constructs based on Self-Determination Theory and the behavioral outcomes of exercise and physical activity revealed a positive association between more autonomous forms of motivation and exercise [48]. Furthermore, competence satisfaction and more intrinsic motives positively predict exercise involvement in several samples [48]. In a meta-analysis of randomized clinical trials, it was possible to identify significant albeit small effects for physical activity, sedentary behavior, diet, alcohol consumption, and smoking cessation [49]. Another study, developed in Norway, examined, based on Self-Determination Theory, how maladaptive motivational processes at work are related to sleep disorders and mental health problems [50]. The findings indicated that participants were more likely to report sleep disturbances, anxiety, and depressive symptoms when feeling frustrated in relation to basic psychological needs for autonomy, competence, and relatedness in the workplace [50]. In other words, the stronger the psychological needs for a given behavior, the more robust the self-determined motivation to perform it. However, there is a gap in the literature concerning the applicability of the Self-Determination Theory in clinical trials specifically targeting 24-h movement behaviors in adults with depressive symptoms. Additionally, there is a need to investigate autonomy, competence, and relatedness as potential mediators associated with the effectiveness of these interventions.

More recently, an expert consensus study [36] and a meta-analysis [37] were carried out in order to identify and classify techniques used to promote the satisfaction and motivation of basic psychological needs in health interventions supported by Self-Determination Theory. This is because, although the effects found from these interventions are effective in motivating the adoption and maintenance of health-related behaviors, as well as promoting adaptive psychological outcomes, there is still considerable variability in the application of the theory in these contexts [36, 37]. Teixeira and colleagues [36] developed a final classification comprising 21 motivation and behavior change techniques, with each technique organized according to the most closely related construct of satisfying psychological needs. The authors also highlighted “a considerable interrelatedness among the motivation and behavior change techniques and the underlying constructs they are proposed to change” [36]. Gillison et al. [37], based on the analysis of 74 intervention studies, identified that the techniques currently in use had the potential to cause changes in the theoretical mediators of health behavior, with a large effect size for autonomy and support for autonomy, moderate for the need for competence, and small for relatedness and motivation. Greater competence satisfaction was observed in individual interventions, when compared to group interventions, and for adults than for children [37]. The results and discussions presented provide support to (a) improve the consistency in the descriptions of interventions based on the Self-Determination Theory, providing social agents, whether researchers, interventionists, or health professionals, with a set of pre-established terms, definitions, and comprehensive approaches that facilitate understanding and precision of the approaches used [36, 37]; and (b) conduct more expressive and pertinent comparisons of interventions based on the components they incorporate, enabling the identification of the most promising techniques for achieving more effective behavior changes [51].

From the content presented, it is evident that the Self-Determination Theory can help to better understand the reasons that lead individuals to adopt to and maintain health-related behaviors, making it a valuable tool for interventions in this context. The premise is that fulfilling basic psychological needs can enhance intrinsic motivation, thereby influencing behavior change. Thus, according to the creators of the Self-Determination Theory, “[…] research on the conditions that foster versus undermine positive human potentials has both theoretical import and practical significance because it can contribute not only to formal knowledge of the causes of human behavior but also to the design of social environments that optimize people’s development, performance, and well-being” ([32, 33], p.68).

### Experimental procedure

**Fig. 2:**
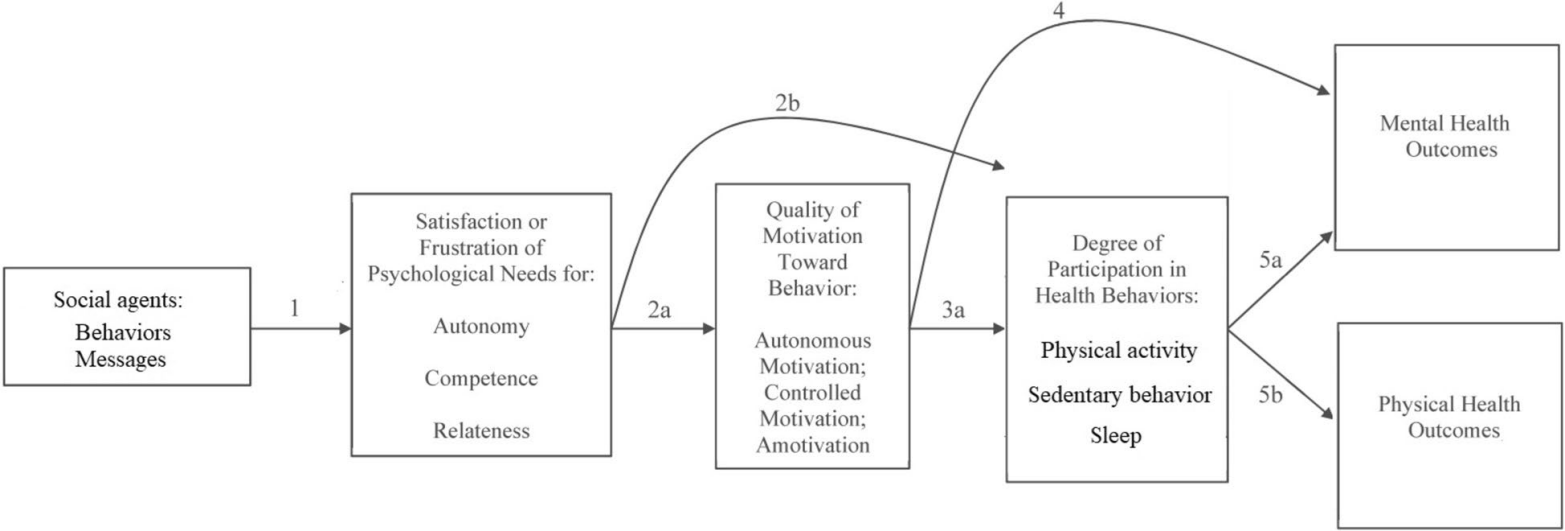
Basic process model of Self-Determination Theory developed for an intervention program. Source: adapted from Teixeira et al. [36]

The intervention was carried out twice a week, on alternate days, spanning a total duration of 16 weeks. To enhance communication and enable active participation in meetings, promoting conversational dynamics and practical engagement, the IG participants were divided into two groups. The sessions, lasting approximately one and a half hours, were conducted both in person (80%) and online (20%), using the Google Meet platform. All meetings were based on the Self-Determination Theory and involved activities conducted by professionals in the field of Physical Education, affiliated with UFSC, along with the participation of external guests. These professionals were tasked, through the proposed dynamics, to support the basic psychological needs of the participants. This influence on the quality of motivation for behaviors and the level of participation/involvement in these behaviors has the potential to bring about changes in mental and physical health outcomes. In all meetings, professionals sought to implement the classification system of techniques constituting interventions based on Self-Determination Theory, as developed by Teixeira et al. [36]. The organizing principle of this system revolves around the satisfaction of psychological needs within healthcare contexts. According to the authors, each technique targeted the construct most closely related to psychological need satisfaction (autonomy, competence, or relatedness), with the aim of achieving a heightened level of motivation and promoting behavior change. In Fig. 2, it is possible to visualize the basic process model of the Self-Determination Theory that was developed in the intervention program.

The actions aimed to enhance participants’ awareness of their living conditions and health, delving into 24-h movement behaviors, especially physical activity, and their associations with depressive symptoms. As mentioned previously, all actions sought to implement techniques to support basic psychological needs and, in specific interventions, gamification strategies were employed. These strategies consist of mechanisms designed to motivate individuals to take action, aid in problem-solving, and facilitate learning [52]. This method has been used in educational programs [52] and coaching, demonstrating its efficacy in bringing about significant changes when integrated into daily life circumstances [53]. To this end, challenges/games were proposed, in which participants had to complete them until the subsequent meeting and present them to the person overseeing the dynamics. The psychology behind gamification seeks to stimulate learning, promote overcoming challenges, and motivate behaviors with the aim of recognizing those with better performance. It is important to highlight that this process must rely on voluntary participation, with all participants being aware of the rules and objectives of the task. Furthermore, the three components of the Unifying Theory of Physical Activity, developed and recently published by Matias and Piggin [54], were taken into account. The first component consists of the idea that physical activity/human movement is the result of inherent impulses, such as feeling, exploring, transforming and connecting, which “contribute to the meanings and purposes that sustain life and growth.” The intermediate level corresponds to the conditions/characteristics of physical activity, considering it as potential, distinct, and integrated. Finally, at the most external level, there are the social, political, and contextual qualities/forces that “interplay with the urges and shape human experience in/of physical activity” [54].

The main activities included (a) lectures covering various topics focused on the adoption and maintenance of healthy habits; (b) distribution of educational materials, including instruction manuals, addressing the necessary care individuals should take in their daily lives; (c) group discussions aimed at exploring the development and progress of the intervention; (d) practical activities involving the participation of family and friends; and (e) practical classes, such as yoga, capoeira, exergames, cycling, walking with pets, weight training, functional training, and gymnastics. The meetings followed a schedule with specific dates, times, and content, divided into four blocks, each consisting of eight sessions. The blocks were organized as follows: (a) contextualization, involving moments for the group to better acquaint themselves and dynamic activities addressing topics such as the relationship between depressive symptoms and 24-h movement behaviors; (b) adherence, providing general and specific guidelines on 24-h movement behaviors; (c) experimentation, offering participants more opportunities to engage in physical activity in different contexts and situations; and (d) maintenance, focusing on strategies and tools for maintaining healthy behaviors in both the short and long term.

Face-to-face meetings were conducted at the onset of each block, which represented 4 weeks of intervention, in order to establish a connection among participants and fostering discussions about their experiences during these periods. The face-to-face meetings took place at the premises of the Federal University of Santa Catarina and at locations suitable for engaging in physical activities. The remaining sessions were conducted via an online meeting platform. A WhatsApp group was also created to address inquiries, distribute support materials, and share photos, audios, and/or other resources related to the proposed activities, addressing other demands that emerged throughout the program. Tables 1, 2, 3, and 4 present the themes, objectives, support techniques for basic psychological needs, and the approaches/actions implemented in each meeting throughout the program, with each table corresponding to 1 month (4 weeks of intervention). In the supplementary material, it is possible to check the lesson plans for all 32 meetings of the Vincular Project, providing detailed information about the proposed content and dynamics.

**Table 1:** Themes, objectives, dynamics carried out, explored psychological needs, and intervention strategies (week 1 to week 4)

Theme (references)	Objectives	Class dynamics	Take-home dynamics	Psychological needs	Intervention strategies for psychological needs
Meeting1: Introductory lesson □ [1]	To present the program schedule and meet the participants	- Presentation: the speaker throws a ball to a random person, who has to introduce themselves. The person who has just introduced themselves throws the ball to someone else, and so on	- Dynamics of similarities: in pairs or trios, the participants should talk with the aim of getting to know each other a little better and finding similarities and differences	Relatedness: involvement and connection	- Considering knowing the participants’ life story - Engaging actively in activities, including doing and playing together - Valuing group activities
Meeting 2: Depressive symptoms □ [2, 3]	To talk about depressive symptoms, their determinants and coping possibilities	- Report on the dynamics of similarities - "Breaking sedentary behavior". Participants stand in a circle. A rope is placed in the middle of the circle and some depressive symptoms are mentioned, as well as common situations among those who suffer from depressive symptoms. Those who perceive the symptoms and situations mentioned should approach the rope	- Do some physical activity of the participants’ own choosing	Autonomy: provision of justification Autonomy: orientation with intrinsic objective Competence: promotion of education	- Reflecting on reasons (internal and external) for adhering to the programs of physical activity - Tracing short-term goals - Providing educational material containing multidisciplinary information pertinent to the physical activity and health relationship
Meeting 3: Introduction to physical activity ○ [4, 5]	To explore the concept of physical activity, its benefits, domains, most practiced types and its relationship with depression	- Participants’ account of their chosen physical activity - "Do you practice physical activity?". Participants are asked to vote on whether they do any physical activity - "What are the benefits of physical activity?". Through an online platform, participants must report the benefits they know about the regular practice of physical activity and these will appear in a "word cloud" format	- Observe patterns of sedentary behavior and sleep (duration and quality)	Autonomy: language style Competence: provision of challenge	- Ensuring that the discourse is not mandatory, that ensures choice about what to do, such as different types of exercises, about how to adapt the activities - Situations that culminate in gamified processes
Meeting 4: Introduction to sedentary behavior and sleep ○ [3, 6]	To explore the concept of sedentary behavior, its harms, domains and most common types. Address the concept of sleep, its stages and sleep hygiene techniques	- Discussion on the patterns observed - "What can we do to break sedentary behavior?". Participants should list strategies or tips on how to reduce sedentary behavior - "How can we get a better quality of sleep?". Participants should list strategies or tips related to sleep hygiene	- Think about general questions related to physical activity and send them to the professors	Autonomy: language style Competence: provision of challenge	- Ensuring that the discourse is not mandatory, that ensures choice about what to do, such as different types of exercises, about how to adapt the activities - Situations that culminate in gamified processes
Meeting 5: Health-related physical fitness □ [7, 8]	To explore the elements of health-related physical fitness	Practice with different stations: - Warm-up: "10 passes" - Cardiorespiratory fitness: running and jumping jacks - Strength: squat and push-up - Agility: "boss sent" - Dynamic balance and coordination - Static balance - Flexibility	- For those who haven't done it yet, think about and list general doubts related to physical activity	Autonomy: structural facilitation Competence: provision of encouragement and support	- Adjusting the level of demand for the participants’ capacity to respond to the task - Adapting and building environmental alterations that ensure the beginning of the activity - Providing positive feedback to the class and individually, whether recognizing an effort of the participant or complimenting his attitude
Meeting 6: Barriers and facilitators to physical activity ○ [9]	To contribute to new reflections on the barriers and facilitators to physical activity	- Using the Mentimeter platform, identify the main barriers to physical activity perceived by the participants and discuss them, seeking to explore the frequency with which the barriers are perceived and the main reasons for this	- Family invitation: for the holiday, the participant should call a friend or family member to do some physical activity and send a record (audio, video, photo, text) about the experience	Competence: facilitation for completing processes relative to goals Relatedness: social support	- Different conditioning forms of achieving the goals - Encouraging the participant to invite friends and Family to carry out/participant in the programs of physical activity
Meeting 7: Curiosities about practicing physical activity ○	To propose a question and answer class on the world of physical activity. With all the questions sent in by the participants, the "Cards on the table" dynamic will be created. Each card has a question or a challenge, such as performing some physical activity. The participant must choose a number and, when the card is revealed, read it out loud to everyone. If the card chosen is a question, the participants can talk to each other in an attempt to answer the question together. If they don't know the answer, the teachers in charge help with the dynamic. If the letter chosen is a challenge, everyone should do it together		-	Autonomy: language style Competence: promotion of education	- Opting for language that ensures choice about what to do, such as different types of exercise, about how to adapt the activities - Providing educational material containing multidisciplinary information pertinent to the physical activity and health relationship
Meeting 8: Lecture with Laine Valgas □	To attend the lecture "Awaken the incredible in me"	- Conversation - Dance - Emotional support - Dynamics in pairs	- Reflection questions, such as why he is grateful for today, what he has learned this day, what he has done for others and what he has done for himself	Competence: provision of feedback	- Recognizing the improvement reached by the participant and remembering the “path” taken to reach it

□: face-to-face meetings; ○: online meetings; 1: Gillison et al. [37]; 2: Pearce et al. [10]; 3: Zhai, Zhang, Zhang [21]; 4: Nahas [55]; 5: Teychenne et al. [56]; 6: Hallgren et al. [57, 58]; 7: American College of Sports Medicine [59]; 8: Garber et al. [60]; 9: Rech et al. [61]

**Table 2:** Themes, objectives, dynamics carried out, explored psychological needs, and intervention strategies (week 5 to week 8)

Theme (references)	Objectives	Class dynamics	Take-home dynamics	Psychological needs	Intervention strategies for psychological needs
Meeting 9: Conducting the intervention □ [1]	To discuss the experiences of the first month	- "Knot dynamics". The participants stand in a circle and memorize who is on their left and right side. To the sound of music, they walk freely around the room. When the teacher asks them to, they must stop where they are and hold hands with the people next to them, but without moving. The aim is to undo the knot and return to the starting position	- Fill in a list of routine activities in order to assess how they can better optimize their time and adopt new healthy behaviors in the short and long term	Competence: barriers acknowledge Relatedness: social support	- Recognizing the stages for behavior chances of each participant - Encouraging the group`s cohesion in the activities and resolution of problems
Meeting 10: Tools for 24-h movement patterns ○ [2–5]	To provide information on resources that help promote physical activity, reduce sedentary behavior and improve sleep quality	- Practicing physical activity and meditation through YouTube channels	- Choose two apps or another tool, try them out and report back on the experience. If possible, invite someone to practice together	Autonomy: provision of choice Relatedness: social support	- Favoring that the participant has and perceives option of choice in the conduction of all stages relative to the program of physical activity - Encouraging the participant to invite friends and Family to carry out/participant in the programs of physical activity
Meeting 11: *Exergames* □ [6–8]	To provide and explore a new tool for practicing physical activity, using all the elements of the body culture of movement	- Practice with Xbox 360: Just Dance, Kinect Sports	-	Relatedness: involvement and connection	- Engaging actively in activities, including doing and playing together
Meeting 12: Lecture with Duda Werner □	To attend the lecture "Short and firm steps: an invitation to self-care with compassion"	- "Presentation dynamics". In order to value the individual for who they are and what they like, they must not define themselves from their professional life, but make a gentle and affectionate presentation, taking into account other aspects and spheres of life - "Dream list". A moment for each participant to reflect on their short-, medium- and long-term dreams	- "Box of happiness". The participant should list on a piece of paper various activities, things they like to do and that make them happy, cut out each one and keep them in a box. When they're having a bad day or realize they need to celebrate an achievement, they can open the box, take out a piece of paper and do what it says	Competence: facilitation for completing processes relative to goals	- Centering goals in the process - Valuing self-comparison as opposed to comparison with peers
Meeting 13: Environment, physical activity and their possibilities □ [9–12]	To explore and recognize a public space and discover the diversity of physical activity practices	- Recognizing space based on characteristics related to resources, conditions, access, aesthetics and safety - Human tic-tac-toe - Playing with a hula hoop - Steal tail - Flag-picking - Talking about collective vs. individual activities, outdoors vs. indoors, recreational vs. competitive, with vs. without supervision	- Recognizing the area in which they live (observing the place, the people who frequent it, what time of day it is busiest and what the built and perceived environment is like)	Autonomy: structural facilitation Autonomy: recognition of the participants’ perspective Competence: provision of challenges	- Recognizing the environmental potential for exercising by ensuring multiple potentials - Adapting and building environmental alterations that ensure the beginning of the activity - Considering participants’ reasons to exercise, such as preferences, expectations about health outcomes, and the cognitive and affective relationships established in the context of physical activity - Creates challenges to break the routine of the exercise sessions, whether for carrying out an exercise in a more challenging manner or carrying out activities in other environments
Meeting 14: Capoeira □ [13–16]	To learn about the history of capoeira, its institutionalization process and its main aspects, and practice it	- Practical initiation exercises - Activities with the basic elements and methodological aspects of capoeira teaching	-	Competence: provision of challenges Relatedness: Involvement and connection	- Creates challenges to break the routine of the exercise sessions, whether for carrying out an exercise in a more challenging manner or carrying out activities in other environments - Valuing group activities
Meeting 15: Les Mills □ [17–19]	To provide more vigorous forms of physical activity	- Power jump - Bodyattack	-	Autonomy: provision of choice	- Favoring that the participant has and perceives option of choice in the conduction of all stages relative to the program of physical activity
Meeting 16: Volleyball □ [20–22]	To propose a collective activity with the ball, recognizing and executing the main technical fundamentals of volleyball	- Warm-up games - Doubles exercises (set, bump, and ball control) - Mini court exercises - Game	-	Competence: provision of encouragement and support Relatedness: involvement and connection	- Providing positive feedback to the class and individually, whether recognizing an effort of the participant or complimenting his attitude - Valuing group activities

□: face-to-face meetings; ○: online meetings; 1: Gillison et al. [37]; 2: Aldenaini et al. [62]; 3: Pradal-Cano et al. [63]; 4: Rodríguez-González et al. [64]; 5: Silva et al. [65]; 6: Cugusi, Prosperini, Mura [66]; 7: Huang et al. [67]; 8: Li, Theng, Foo [68]; 9: Ferrari et al. [69]; 10: Kowitt et al. [70]; 11: Pontin et al. [71]; 12: Stappers et al. [72]; 13: Amitay [73]; 14: Delattre; Collaer [74]; 15: Jordan et al. [75]; 16: Martins et al. [76]; 17: Cunha [77]; 18: Jones et al. [78]; 19: Zureigat et al. [79]; 20: Guo et al. [80]; 21: Mohammadi [81]; 22: Vaccaro et al. [82]

**Table 3:** Themes, objectives, dynamics carried out, explored psychological needs, and intervention strategies (week 9 to week 12)

Theme (references)	Objectives	Class dynamics	Take-home dynamics	Psychological needs	Intervention strategies for psychological needs
Meeting 17: Conducting the intervention □ [1]	To discuss the positive and negative aspects of the intervention and behavioral changes	- "Compliments board". Knowing that participants have difficulty identifying and recognizing their own qualities, each participant should praise their colleagues, creating a repertoire of positive characteristics of each one	-	Autonomy: emphasis on responsibility Competence: provision of feedback	- Raising the participants’ awareness about the need for advancing in the dynamics involved in the program of physical activities - Reflect with participants on aspects that still need improvement, visualize how to proceed/continue
Meeting 18: Weight training □ [2–5]	To propose a resistance training class, using resources available in the gym (weights and machines)	- Joint warm-up - Dumbbell bench press - Low row - Lateral raise - Dumbbell curl - Triceps on pulley - 45º leg press - Extension and flexor chair - Adductor and abductor chair - Calf raises	- Perform sets of abdominal crunches according to the teacher's instructions	Autonomy: provision of choice Relatedness: involvement and connection	- Favoring that the participant has and perceives option of choice in the conduction of all stages relative to the program of physical activity - Engaging actively in activities, including doing and playing together
Meeting 19: Functional training □ [6, 7]	To offer a meeting focused on activities related to control, stability and motor coordination, contributing to the performance of usual activities	Practice in circuit format: - Joint warm-up - Climber + jump on bench - Abdominal rowing - Support/push-up - Pull-up/inverted crucifix - Dips + lateral raises - Combined exercise - Plyometric - Abdominal plank - Coordination + squat - Juggling + jump rope	-	Autonomy: structural facilitation Competence: facilitation for completing processes relative to goals	- Recognizing the environmental potential for exercising by ensuring multiple potentials - Different conditioning forms of achieving the goals
Meeting 20: Dance □ [8, 9]	To experience different dance practices	- Dance initiation, with samba no pé, samba de gafieira and zouk	-	Competence: provision of challenges	- Creates challenges to break the routine of the exercise sessions, whether for carrying out an exercise in a more challenging manner or carrying out activities in other environments
Meeting 21: Yoga □ [10–13]	To try the basic yoga positions and do a relaxation practice	- Beginners' practice and breathing exercises	-	Autonomy: structural facilitation Competence: provision of encouragement and support	- Adjusting the level of demand for the participants’ capacity to respond to the task - Providing positive feedback to the class and individually, whether recognizing an effort of the participant or complimenting his attitude
Meeting 22: *Slackline* □ [14–16]	To work with different forms of balance through the practice of slackline	- Stretching exercises, individually and in pairs - Balance exercises with bosu and balance platform - Slackline practice, with individual exercises, in pairs, in trios and with everyone	-	Autonomy: orientation with intrinsic objective	- Rationalizing the role of physical activity for reasons such as building a sense of friendship, improvement of skills, energy gain, and a better lifestyle
Meeting 23: Cycling tour □ [14–17]	To practice in groups and outdoors	- Route from UFSC to the seafront	-	Relatedness: involvement and connection	- Valuing group activities
Meeting 24: Sand sports □ [14–16]	To provide a meeting where participants have the opportunity to try beach tennis	Practice at Arena Beach Floripa: - Warm-up - Ball handling drills - Service drills - Moving drills - Mini game	-	Autonomy: structural facilitation Relatedness: cooperation group	- Adjusting the level of demand for the participants’ capacity to respond to the task - Creating groups in virtual environments for experience exchange, facilitating the communication between participants

□: face-to-face meetings; ○: online meetings; 1: Gillison et al. [37]; 2: Bennie et al. [83]; 3: Carneiro et al. [84]; 4: Gordon et al. [85]; 5: Marques et al. [86]; 6: American College of Sports Medicine [59]; 7: Garber et al. [60]; 8: Hellem et al. [87]; 9: Karkou et al. [88]; 10: Breedvelt et al. [89]; 11: Brinsley et al. [90]; 12: Cramer et al. [91] 13: Nanthakumar [92]; 14: Brito et al. [93]; 15: Coventry et al. [94]; 16: Frühauf et al. [95]; 17: Matias et al. [54, 96]

**Table 4:** Themes, objectives, dynamics carried out, explored psychological needs, and intervention strategies (week 13 to week 16)

Theme (references)	Objectives	Class dynamics	Take-home dynamics	Psychological needs	Intervention strategies for psychological needs
Meeting 25: Conducting the intervention □ [1]	To discuss the positive and negative aspects of the intervention	- As a form of social interaction, messages are projected in video format, recorded by the participants’ family members, telling a little more about the participant and their perceptions of Vincular Project in the participants’ life	-	Autonomy: provision of justification Autonomy: orientation with intrinsic objective Competence: provision of feedback	- Assessing and reassessing long-term aims - Recognizing internal reasons relative to physical activity, such as satisfaction and pleasure - Recognizing the improvement reached by the participant and remembering the “path” taken to reach it
Meeting 26: Pet day □ [2–4]	To discuss the relationship between animals and people living with depressive symptoms	- Walk with participants’ pets	-	Relatedness: social support Relatedness: involvement and connection	- Encouraging the participant to invite friends and family to carry out/participant in the programs of physical activity - Valuing group activities
Meeting 27: Workers' quality of life and active ageing ○ [5]	To cover content such as assessing the quality of life of the worker, the profile of the work environment and considerations and the importance of leisure, as well as content on guidance for an active lifestyle in old age, maintaining good habits throughout life	- Conversation round	- Fill in the "Well-being Pentacle" and the "Work Environment and Conditions Profile" tools	Autonomy: recognition of the participants’ perspective	- Considering participants’ reasons to exercise, such as preferences, expectations about health outcomes, and the cognitive and affective relationships established in the context of physical activity
Meeting 28: Labour gymnastics □ [6–8]	To talk about the concept and benefits of occupational gymnastics and carrying out different practices	- Stretching practice - Group dynamics - Self-massage and guided relaxation	-	Autonomy: language style	- Opting for language that ensures choice about what to do, such as different types of exercise, about how to adapt the activities
Meeting 29: Talk with Gabriela Cunha ○ [9–11]	To attend the lecture "Quality of life and eating behavior: learning to deal with emotional eating"	- Chat session, with moments of reflection and awareness on the subject of quality of life and eating behavior	- Think about collective activities for the gymkhana	Autonomy: provision of choice Competence: promotion for education	- Favoring that the participant has and perceives option of choice in the conduction of all stages relative to the program of physical activity - Providing educational material containing multidisciplinary information pertinent to the physical activity and health relationship
Meeting 30: Gymnastics for everyone □ [12–14]	To propose gymnastic activities that take into account the different types of bodily manifestations	- Beginners' practices		Autonomy: structural facilitation Competence: provision of encouragement and support	- Adjusting the level of demand for the participants’ capacity to respond to the task - Providing positive feedback to the class and individually, whether recognizing an effort of the participant or complimenting his attitude
Meeting 31: Gymkhana □ [15–17]	To explore collective and competitive activities through activities and games with family and friends	- Equal numbers - Three-legged race - Egg on spoon race - Unscrambling the letters - Emperor penguin - Bridge with hula hoops - Target shooting + balloon popping	-	Competence: facilitation for completing processes relative to goals Relatedness: social support	- Different conditioning forms of achieving the goals - Creating situations that essentially culminate in the participation of family and friends in activities - Encouraging the group`s cohesion in the activities and resolution of problems
Meeting 32: Project closing: trail □ [15–17]	To provide moments of reflection on the trajectory of the intervention and future prospects	Trail on the Costa da Lagoa (Florianópolis/SC)	- Send a statement about your experience with the project, with your perceptions of the issues addressed in the intervention	Autonomy: orientation with intrinsic objective Competence: provision of encouragement and support Relatedness: cooperation group	- Rationalizing the role of physical activity for reasons such as building a sense of friendship, improvement of skills, energy gain, and a better lifestyle - providing positive feedback to the class and individually, whether recognizing an effort of the participant or complimenting his attitude - Creating groups in virtual environments for experience exchange, facilitating the communication between participants

□: face-to-face meetings; ○: online meetings; 1: Gillison et al. [37]; 2: Brooks et al. [97]; 3: Friedman; Krause-Parello [98]; 4: Kamioka et al. [99]; 5: Nahas [55]; 6: Conn et al. [100]; 7: Laux et al. [101]; 8: Serra; Pimenta; Quemelo [102]; 9: Ekinci; Sanlier [103]; 10: Kris-Etherton et al. [104]; 11: Singh et al. [105]; 12: Bento-Soares; Schiavon [106]; 13: Menegaldo; Bortoleto; Mateu [107]; 14: Menegaldo; Bortoleto [108]; 15: Brito et al. [93]; 16: Coventry et al. [94﻿]; 17: Frühauf et al. [95]

The GC, in turn, was expected to continue with its usual activities. However, recognizing the high chance of participants allocated to this group dropping out the research, a decision was made to maintain contact through telephone messages every 2 weeks. The content of these messages included psychoeducational materials on mental health, as well as guidelines for practicing physical activity, reducing sedentary behavior, and improving sleep, which were also shared with individuals who did not meet the eligibility criteria, as mentioned previously (Table 5). It is also worth noting that, if the effectiveness of the intervention is confirmed, the researchers plan to offer the intervention to the control group later, with the goal of extending benefits to all study participants.

**Table 5:** Support materials provided to control group participants

Materials	Description	Access
• Physical activity guide for the Brazilian population	• This document presents the first recommendations on physical activity across all life stages, including specific guidelines for pregnant women and individuals with disabilities, tailored to the Brazilian population • It outlines strategies aimed at encouraging a more active lifestyle among the population, thereby promoting health and enhancing quality of life	•https://bvsms.saude.gov.br/bvs/publicacoes/guia_atividade_fisica_populacao_brasileira.pdf
• Support information • "I had a black dog, his name was depression" video	• Contact information for Basic Health Units, Psychosocial Care Centers, facilities offering free or subsidized psychological care in Florianópolis, and other available services • An educational video/short film produced by the World Health Organization, addressing various issues related to depression	•https://sapsi.ufsc.br/files/2021/08/Rede-de-Apoio-SAPSI-7.pdf •https://www.youtube.com/watch?v=XiCrniLQGYc&t=5s
• "Depression: when knowing how to talk and listen inspires life" document	• Created by Janssen and Johnson & Johnson, this material is intended for those who believe that "empathy and acceptance can save lives and make society more humane". The guide addresses several key topics about depression that everyone should be aware of	•https://biblioteca.cofen.gov.br/wp-content/uploads/2021/06/depressao-quando-saber-falar-e-ouvir-inspira-a-vida.pdf
• "How to see the most invisible side of health" video • "The benefits of non-traditional exercises" video	• Videos from the "Autoridade Fitness" channel, available on YouTube • The first video highlights the importance of treating mental health with the same care we give to physical health • The second video offers examples of activities that go beyond the typical gym or weight training routines	•https://www.youtube.com/watch?v=9Asrl9_6FQQ •https://www.youtube.com/watch?v=DsWW9ct17WA
• "WHO guidelines on physical activity and sedentary behaviour" • "Why do cell phones cause significant harm to your spine?" video	• Manual with recommendations on the amount of physical activity (frequency, intensity and duration) required to offer significant health benefits and mitigate health risks • Video from the "Autoridade Fitness" channel, available on YouTube	•https://iris.who.int/bitstream/handle/10665/336656/9789240015128-eng.pdf?sequence=1 •https://www.youtube.com/watch?v=GuCXqBFRiv4
• Sleep hygiene tips • Booklet with guidelines on sleep hygiene • "The danger of lack of sleep in nutrition" video	• Material prepared by the Integrated Health System (SIS) offering tips on sleep hygiene • Booklet with guidelines on sleep hygiene. This material was developed by the Federal Rural University of the Amazon (UFRA) • Video from the "Autoridade Fitness" channel, available on YouTube	•https://www12.senado.leg.br/institucional/sis/noticias-comum/higiene-do-sono-saiba-o-que-e-e-como-ela-pode-melhorar-a-sua-vida •https://repositorio.ufra.edu.br/jspui/bitstream/123456789/1614/1/Cartilha%20%e2%80%9cHigiene%20do%20Sono%e2%80%9d.pdf •https://www.youtube.com/watch?v=Dcl8fjInGjU
• Activity and sleep report • Report interpretation manual	• Report with individual results of accelerometer use, measuring 24-h movement behaviors (physical activity, sedentary behavior and sleep) • Tutorial for interpreting the results of: a) accelerometer usage time; b) moderate to vigorous intensity physical activity; c) total physical activity; d) sleep period; e) sleep efficiency; f) other behavior classifications throughout the day	Unavailable

### Data collection instruments

The initial screening was carried out through an online questionnaire, created on the Google Form® platform, which included basic information to verify the eligibility criteria, as well as application of the Patient Health Questionnaire-9 (PHQ-9) and other screening tools to identify: (a) the need for specialized psychiatric treatment (e.g., psychosis, schizophrenia); (b) the use of antidepressant medication; (c) the requirement for specific treatments targeting depression. This comprehensive approach aimed to efficiently evaluate participants and determine appropriate courses of action based on their responses. The PHQ-9 is designed to evaluate the presence of depressive symptoms through nine questions that address the following aspects: (a) depressed mood; (b) anhedonia (loss of interest or pleasure in doing activities in general); (c) sleep problems; (d) tiredness or lack of energy; (e) change in appetite or weight; (f) feelings of guilt or worthlessness; (g) concentration problems; (h) feelings of sluggishness or restlessness; and (i) suicidal thoughts. The frequency of each symptom in the last 2 weeks is evaluated using a Likert scale ranging from 0 to 3, corresponding to the answers “not at all,” “less than a week,” “a week or more,” and “almost every day,” respectively. Upon completion, the instrument provides a disease severity score from 0 to 27. The questionnaire also includes a tenth question, which, although not contributing to the final score, evaluates the patient’s overall impression of how symptoms impair daily activities, such as work and study, and being associated with the severity of psychiatric symptoms. The validation of this instrument for adults in the general population of Brazil was carried out by Santos and collaborators [109], using a cutoff point of ≥ 9 points to indicate the presence of depression.

Eligible participants subsequently completed a second questionnaire, which incorporated various tools. Although this compilation of instruments was administered online through the Google Form® platform, participants were requested to complete it in the presence of the Vincular Project team, in order to provide assistance when necessary. Some variables were collected using the questionnaire employed in Vigitel telephone surveys, which stands for the Surveillance of Risk and Protective Factors for Chronic Diseases by Telephone Survey, such as (a) sociodemographic information (age, sex, marital status, skin color, and education); (b) behavioral aspects (level of physical activity during leisure time, commuting, domestic, and work); (c) health conditions (from the diagnosis of chronic non-communicable diseases); and (d) self-perceived health. This material was created based on other tools applied by monitoring systems for risk factors for chronic diseases [110, 111] and experiences from system implementation tests carried out in some Brazilian cities [112–114].

The Simple Physical Activity Questionnaire (SIMPAQ), developed and validated by a multidisciplinary group as a clinical instrument to assess physical activity and sedentary behavior in individuals with mental illnesses, was also used [115]. SIMPAQ considers the respondent’s activity over the last 7 days and is structured into five domains, including time spent (a) in bed; (b) in sedentary behavior; (c) walking; (d) doing exercises; and (e) in other activities [115]. Furthermore, another questionnaire was employed to assess the time spent in sedentary behaviors during a typical day of the week and on Sundays in different contexts, such as (a) watching television; (b) using the computer at home; (c) using a cell phone; (d) sitting at work; (e) sitting at school/university; and (f) sitting while traveling by car, motorcycle, or bus [116]. For sleep assessment, the Pittsburgh Sleep Quality Index (PSQI), validated in the adult population of Brazil by Bertolazi et al. [117], was utilized. This instrument evaluates sleep quality over the past month and comprises nine questions, encompassing seven components: (a) subjective sleep quality; (b) sleep latency; (c) sleep duration; (d) habitual sleep efficiency; (e) sleep disorders; (f) use of sleeping medication; and (g) drowsiness and daytime dysfunction. The response options yield a score ranging from 0 to 3, resulting in a total sum of 0 to 21 points. The questionnaire scores can be analyzed as a discrete quantitative variable, with higher scores indicating poorer sleep quality. Alternatively, a cutoff point can be considered, with scores ≤ 5 indicating good sleep quality and scores ≥ 6 signifying poor sleep quality.

For the evaluation of basic psychological needs, the Basic Psychological Needs in Exercise Scale (BPNES), introduced by Vlachopoulos, Ntoumanis, and Smith [118], was utilized. This instrument, translated into Portuguese and adapted for daily activities by Karloh et al. [119], aimed to identify potential changes in the constructs of the Self-Determination Theory following the intervention program. The BPNES is a self-report tool designed to evaluate the extent to which innate needs for autonomy, competence, and relatedness are satisfied in the context of exercise. The questionnaire contains 11 questions with response options ranging from 1 to 5, following the scale “I do not agree,” “I somewhat agree,” “I partially agree,” “I strongly agree,” and “I completely agree,” respectively. The scores are interpreted separately for each basic psychological need, based on the item averages. The perception of barriers to engaging in physical activity was measured using the questionnaire developed by Martins and Petroski [120], which consists of a list of nineteen barriers rated on an ordinal scale, based on the frequency in which the barriers are identified (never = 1; rarely = 2; sometimes = 3; almost always = 4; always = 5). A “perceived barrier” was considered when participants marked the options “always” or “almost always.” Finally, the Fantastic Lifestyle Questionnaire was also administered. Its Brazilian version, translated and validated for young adults, consists of 25 questions divided into nine domains: (a) family and friends; (b) physical activity; (c) nutrition; (d) cigarettes and drugs; (e) alcohol; (f) sleep, seat belts, stress, and safe sex; (g) type of behavior; (h) introspection; and (i) work [121]. The questionnaire employs a Likert scale for answer options. Each question is assigned a specific code, and the sum of points classifies individuals into five categories: (a) excellent (85 to 100 points); (b) very good (70 to 84 points); (c) good (55 to 69 points); (d) regular (35 to 54 points); (e) needs improvement (0 to 34 points) [121].

Participants also used Actigraph GT3X + and wGT3X + accelerometers (ActiGraph Corporation, Pensacola, FL, USA), made available by the Center for Research in Physical Activity and Health (NuPAF), at UFSC, for objective measurement of 24-h movement behaviors. These devices were attached to the non-dominant wrist using an imported PVC bracelet for a continuous 7-day period. Participants followed a 24-h protocol, removing the device only for swimming or water activities involving submersion (but not for other activities with water, such as taking a shower or washing dishes). However, due to the number of devices damaged in the initial collection, participants were instructed to remove the accelerometer during bathing in the second and third collections as a precautionary measure. The data were collected at 30 Hz and the raw acceleration data were utilized. Subsequently, these data underwent a self-calibration process and were converted into the ENMO metric (Euclidean norm minus one), expressed in milligravity units (mg). Further details about the processing of raw accelerometry data can be found in the article by Migueles and colleagues [122]. Thus, the data were analyzed in periods of 5-s epochs and the activities were classified into three categories: (a) sedentary behavior (< 35.6 mg); (b) light physical activity (between 35.6 and 201.4 mg); and (c) moderate to vigorous physical activity (≥ 201.4 mg), adopting the cutoff points of Hildebrand et al. [123, 124]. Sleep duration was estimated using the heuristic algorithm by observing the distribution of change in the Z angle [125]. Participants with at least three valid accelerometer days, comprising two weekdays and one weekend day, each with a minimum duration of 16 h, were included in the analyses. A measurement day was defined as the time interval between wake-up times, accounting for daily cycles that might last more or less than 24 h in total. The accelerometers were programmed and data were downloaded using Actilife software, version 6.8.11, for Windows. Raw data analyses were performed using the GGIR package, version 2.9.5.

The team from Vincular Project was made up of undergraduate, master’s, and doctoral students in the field of physical activity and health. Before the pre-intervention assessment, all team members participated in training to familiarize themselves with the assessment protocols and the application of the questionnaires. All aspects were discussed point by point to ensure procedural standardization. In Table 6, it is possible to verify the schedule of enrolment, interventions, and assessments with details.

**Table 6:**
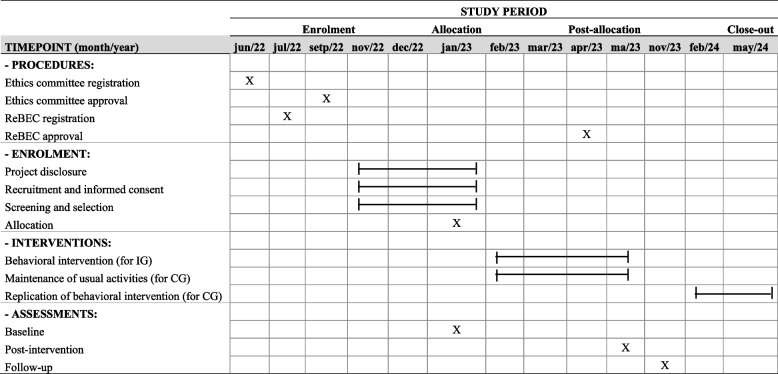
Schedule of enrolment, interventions, and assessments

*IG* intervention group, *CG* control group

### Study variables

The primary outcome of the study was depressive symptoms and secondary outcomes were 24-h movement behaviors (physical activity, sedentary behavior, and sleep) and basic psychological needs (autonomy, competence, and relatedness). Autonomy, competence, and relatedness variables were also examined as potential mediators influencing the effectiveness of the intervention on depressive symptoms. Furthermore, sociodemographic factors, health conditions, self-perceived health, lifestyle, and factors related to the perception of barriers to practicing physical activity were considered as descriptive and/or adjustment variables.

### Data treatment and statistical analyses

The tabulation of questionnaire data is conducted through the Google Form® platform, which automatically generates an Excel spreadsheet as soon as participants respond to the tools. At the conclusion of the process, the tabulation will be checked manually by the Vincular Project team. Database management and processing, as well as descriptive and inferential analyses, will be performed using different statistical packages, including the Statistical Package for the Social Sciences (SPSS, version 23.0) and RStudio (version 2021.09.0).

Continuous variables will be described using measures of central tendency and variability, while categorical variables will be presented in terms of absolute and relative frequencies, along with their respective 95% confidence intervals. Before applying hypothesis tests, the normality of continuous data will be assessed through (a) kurtosis and asymmetry and (b) the Kolmogorov–Smirnov test. In the absence of normality in the data distribution, non-parametric procedures will be adopted. Comparisons of the measured variables between groups at baseline will be conducted using the Student’s t-test for independent samples (or its non-parametric equivalent, the Mann–Whitney U test), and the Chi-Square test for categorical variables.

To verify the effect of the intervention on the primary outcome and secondary outcomes, per-protocol and intention-to-treat analyses will be conducted. The first one will take into account only IG participants who followed the activities until the last meeting, complied with the planned intervention protocol with at least 50% overall attendance and in each of the four blocks of the intervention, in addition to being duly evaluated in the line moments baseline and post-intervention. This decision aligns with the intervention proposal, in which the initial meetings comprised more theoretical activities, with practical sessions beginning in the second month onwards. Those from the GC who participated in the assessments will also be considered. The second analysis will include all individuals from the CG who participated in both data collections, and those from the IG considered in the per-protocol analyses. To fill in the missing data from the other participants, the multiple imputation technique will be applied, using sex, age, and the variables of interest as predictors. For comparisons between periods (pre- and post-intervention) and between groups (intervention and control), analysis will be conducted using generalized estimating equations (GEE), with Bonferroni post hoc adjustments. The significance threshold will be set at *p* ≤ 0.05. However, for time*group interactions, *p* ≤ 0.10 will be considered marginally significant [126]. Additionally, effect sizes will be calculated based on (a) Cohen’s d [127], considering small values (0.20 ≤ d < 0.50), medium values (0.50 ≤ d < 0.80), and large (d ≥ 0.80); and (b) partial eta squared (ηp2), with small (0.02 ≤ ηp2 < 0.13), medium (0.13 ≤ ηp2 < 0.26), and large (ηp2 ≥ 0.26) effect sizes [128]. These procedures will be performed using the Statistical Package for the Social Sciences (SPSS, version 23.0) software.

**Fig. 3:**
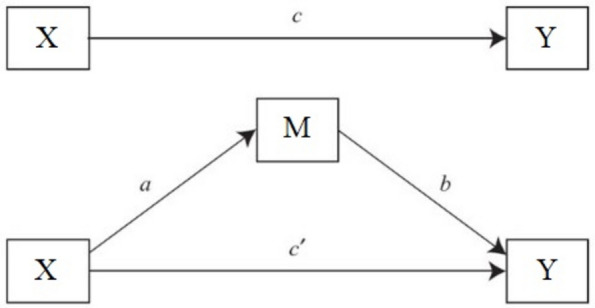
Paths of the mediation model. Source: adapted from Hayes [130]

To investigate the mediating role of basic psychological needs, structural equation modeling procedures will be adopted, using linear regression models, through the lavaan package in the RStudio software (version 2021.09.0). Diagrams will be created using the “Miro” website (https://miro.com). For the structural equation model, the “SEM” command will be utilized with the MLM estimator: maximum likelihood estimation. In this context, the mediating variable corresponds to an intervening factor essential for completing the causal relationship between an independent variable and a target behavior [129]. In intervention studies, a variable is considered a mediator when it alters the positive relationship between the treatment and the investigative variable (exemplified, in the present study, by the CG and IG and by depressive symptoms, respectively). Different analyses can be employed to identify mediating variables, based on statistical procedures that test the hypothesis that an independent variable (treatment) influences a certain outcome (investigative variable) through one or more mediators [130]. For this type of analysis to occur, a causal chain with at least three variables (independent, mediating, and dependent) is necessary, as illustrated in Fig. 3. In this model, the independent variable (treatment) is represented by “X,” while the mediating variable is “M,” and the dependent/investigative variable, “Y.” Thus, the model assumes a system with different possibilities: (a) the treatment impacting the investigative variable, referred to as the total effect (represented by coefficient “c”); (b) the treatment impacting the mediating variable (coefficient “a”); (c) the mediating variable impacting the dependent/investigative variable, with adjustment for the treatment variable (coefficient “b”); and (d) the treatment impacting the investigative variable, adjusting for the mediating variable, known as direct effect (coefficient “c”). The indirect effect of the treatment on the investigative variable through the mediating variable is calculated as the product of coefficients “a” and “b.” The total effect is calculated by summing the direct and indirect effects (c = c’ + ab) [130].

**Fig. 4:**
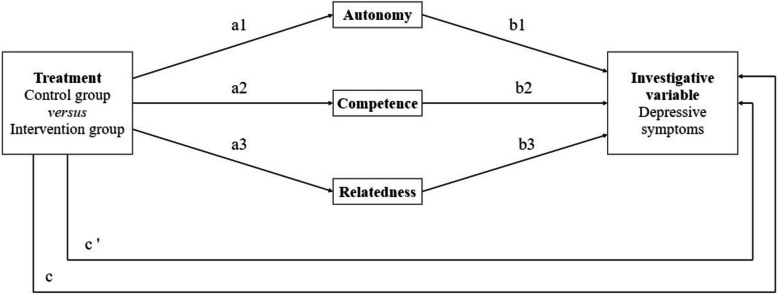
Description of the model of possible mediating variables of the Vincular Project. Source: own authorship (2023). Notes: total effect of the treatment on the investigative variable (coefficient “f”); mediated direct effect of the treatment on the investigative variable (coefficient “f’”); effect of treatment on basic psychological needs (“a” coefficients); effect of the treatment on the investigative variable, including the basic psychological needs variables (coefficients “a” and “b”)

Considering that behavior change interventions aimed at reducing depressive symptoms can involve complex and multidimensional processes, it is unlikely for this outcome to be solely explained by a single mediator. In this context, multiple mediation models will be explored, which simultaneously considering basic psychological needs as potential mediators in the relationship between treatment and depressive symptoms [130, 131], as illustrated in Fig. 4. In this model, the following paths are observed: (a) the total effect of the treatment on the investigative variable (coefficient “f”); (b) the direct effect of the treatment on the investigative variable, with adjustment for the mediating variable (coefficient “f’”); (c) the effect of treatment on basic psychological needs (“a” coefficients); and (d) the effect of the treatment on the investigative variable, including the basic psychological needs variables, which assume the role of mediators (coefficients “a” and “b”).

The variables used in the models will take into account the difference between post-intervention and baseline values. The effect of the intervention on depressive symptoms will be observed directly, obtaining the total direct effect of the intervention. Subsequently, all paths for potential mediating variables will be included to determine the indirect effects of treatment on depressive symptoms. Two models will be tested: the first with the observed variables, represented by the overall PHQ-9 score and the individual autonomy, competence, and relatedness scores; and the second model, considering latent variables, corresponding to each PHQ-9 question and the basic psychological needs instrument. For statistical modeling, the “backward” selection strategy will be adopted, with a critical level of *p* ≤ 0.20 for variables to remain in the model. The following adjustment indices and cutoff points will be utilized: (a) degrees of freedom (df) ≥ 1; (b) chi-square *p*-value (X2) > 0.05; (c) ratio X2 per df < 3; (d) comparative fit index (CFI) and Tucker–Lewis index (TLI) > 0.90; (e) root mean square error of approximation (RMSEA) < 0.08; (f) RMSEA *p*-value < 0.05; and (g) standardized root mean square residual (SRMR) < 0.08 [132].

## Discussion

This paper provides an overview of the Vincular Project, describing the step-by-step development of a behavior change intervention based on Self-Determination Theory to reduce depressive symptoms in Brazilian adults. It is noteworthy that the intervention followed a distinct protocol, with each meeting presenting specific content. The idea of departing from conventional models, such as aerobic and/or muscle-strengthening exercises, and focusing on 24-h movement behaviors, particularly a diverse range of physical activities, is both original and justified. This concept is motivated by the inconsistencies found in the literature concerning the “ideal dose” of these behaviors for the subgroup of interest [56], as well as heterogeneous designs and interventions [21, 28, 57, 58, 133, 134]. Thus, the proposed intervention model seeks to enhance participants’ adherence by prompting reflections on the significance of these behaviors in their lives. The aim is to ensure that, by the end of the protocol, participants possess an expanded perspective on this topic, offering them more choices and experiences capable of energizing other senses and feelings.

Likewise, although the presence of a theoretical contribution regarding the individual associations of movement behaviors with health-related aspects, such as symptoms of depression, is recognized, the inherent codependency between each behavior within a 24-h period is often overlooked [135, 136]. According to Del Pozo Cruz et al. [135], “lifestyle behaviors are compositional in nature.” This means that time for individuals is the same, in which the 24 h of a day can be allocated to a specific behavior, but this will inherently be intertwined with the time spent on other behaviors [135, 136]. Therefore, the implementation of resources addressing multicomponent actions, associated with the integrated management of 24-h movement behaviors, may be a more effective method than traditional approaches that consider behaviors in isolation, in a segmented way, in an attempt to reduce symptoms of depression in the adult population. The potential for a causal relationship between 24-h movement behaviors and mental health also raises a unique perspective; specifically, interventions designed at improving depressive symptoms may simultaneously enhance physical activity, reduce sedentary behavior, and promote better sleep in the adult population.

Another aspect to be considered concerns the evidence of the effectiveness of fundamental interventions based on Self-Determination Theory across a wide range of health domains [37]. However, there is still a gap in the literature regarding the effectiveness of experimental studies focused on actions aimed at 24-h movement behaviors and supported by Self-Determination Theory in reducing depressive symptoms in adults. As an additional factor, the proposal for an intervention grounded in this theory allows professionals to support basic psychological needs (focused on autonomy, competence, and relatedness), thereby fostering autonomous motivation and behavioral change in participants [36]. This approach is highly particular and little explored in clinical trials, addressing intrapersonal, interpersonal, and environmental themes and their relationship with a healthier lifestyle, thus contributing to the non-pharmacological treatment of depression. Interventions that concentrate on a variety of activities can encourage the exploration of intrinsic feelings. This strategy, based on the principles of Self-Determination Theory, facilitates an increase in individual’s motivation and long-term adherence, making studies more pragmatic, with the results obtained lasting immediately after the conclusion of these programs. This becomes relevant considering that individuals with depressive symptoms experience several barriers, such as lack of energy and loss of interest, common factors associated with the disease itself, and which contribute to the abandonment of health promotion in its most varied contexts.

It is also important to highlight the importance of monitoring indicators of depressive symptoms and 24-h movement behaviors after implementing these actions. Depression is associated with several functional disorders and, in some cases, symptoms may recur without appropriate follow-up. Adhering to and maintaining healthy behaviors is a challenge for a significant portion of the population, and this task can become even more difficult for those experiencing depressive symptoms. For the successful management of depression across its three phases—namely the beginning of treatment, continuation, and monitoring—interventions are required to discuss strategies and offer tools for maintaining positive results after the conclusion of this process. This ensures that the benefits persist in people’s daily lives. Moreover, when considering interventions aimed at behavioral changes with a focus on reducing depressive symptoms, it is clear that the satisfaction of basic psychological needs can be considered a key mediator of the expected results [36]. The inclusion of potential mediators in this research is crucial for understanding the mechanisms of change and the elements that can directly or indirectly impact a particular outcome. Information related to this subject favors the understanding of the psychosocial mechanisms involved in modifying behaviors, and consequently, the benefits related to mental health, contributing to improving the design of future interventions in this area. The Vincular Project also aims to achieve other results related to quality of life, such as aspects of health conditions, self-perceived health, and lifestyle. This holistic approach is justified by the need to understand the human being as a whole, complex, and susceptible to influences, while also having an impact on the environment in which they live. This information contributes to a comprehensive perspective on how the intervention can impact the health of adults with depressive symptoms, extending beyond the desired outcomes.

The limitations of this study must be recognized and addressed in future research. Firstly, the inclusion criteria for the study required participants to have electronic devices, such as computers, tablets, or cell phones, and internet access, as some meetings were conducted online. This could introduce a selection bias, given that access to these technologies in low- and middle-income countries, such as Brazil, is a multifaceted issue involving factors like infrastructure, economy, education, and government policies. Another issue is the exclusion of participants who self-reported the need for specialized psychiatric treatment, based on their self-diagnosis, rather than a formal diagnostic assessment. Furthermore, assessments of biochemical markers that could help elucidate the results, along with an examination of the intervention’s implementation and its cost-effectiveness relationship, were not conducted. Finally, it is crucial to underscore the challenge of ensuring that participants in the CG maintain their usual activities and truly represent a pure CG. This difficulty arises because, in the presence of depressive symptoms, individuals may seek alternative treatments or change their behaviors, such as engaging in physical activity. This is a factor beyond the control of research conducted with this format and proposal.

Despite this, it is worth highlighting that this study presents relevant contributions on the effect of behavior change interventions on depression symptoms in adults, contributing to clarifications in the literature, and shedding light on the reality of the subgroup under consideration. The Vincular Project represents one of the first initiatives in a context demanding immediate action and attention. From this perspective, the proposed intervention represents an attempt to promote the implementation of actions, the results of which can provide a foundation for discussing ideas and formulating future public policies. In addition to creating an environment that supports autonomy, competence, and relatedness, the study outlines the specific intervention techniques used, ensuring their quality and replicability. This work goes beyond providing information solely on the overall score of depressive symptoms, offering additional insights into their indicators. Moreover, it delves into 24-h movement behaviors, not just at a global level, but also providing data on their intensity, domains, and types. Another positive aspect of this study is its contribution to a research area that is still under development, particularly in terms of potential mediators related to the effectiveness of interventions on changes in depressive symptoms, an area with limited evidence on the Brazilian scenario.

By creating intervention opportunities, as reported in the present study, which address important variables through multiple approaches, it is possible to educate this specific population group, and offer actions that promote more life in years and not just more years of life. This provides a support network that facilitate feelings such as autonomy, competence, and relatedness. From this perspective, the present study seeks to develop and experiment with mechanisms to promote assertive changes in the lifestyle of adults with depressive symptoms, respecting their individuality and the way they establish their relationships between themselves, their families, and the community.

### Trial status

Concluded. Ethics approval: September 2022. Recruitment: November 2022 to January 2023. Allocation: January 2023. The assessment of the variables of interest was carried out at three moments: (a) baseline pre-intervention (January 2023); (b) post-intervention, immediately following the conclusion of the program (May 2023); and (c) post-intervention, 6 months after the end of the intervention (follow-up evaluation, conducted in November 2023). The authors acknowledge the importance of submitting the protocol before the start of recruitment, in accordance with clinical trial guidelines. However, conducting research in Brazil presents significant operational challenges due to limited investment and lack of external funding opportunities. This study did not receive financial support, which prevented the hiring of professionals to conduct interventions involving complex and specialized content related to human movement. Additionally, the research team was restricted, consisting of only two researchers—one master’s and one doctoral student—under the supervision of a single supervisor. Furthermore, the present study does not represent a conventional physical activity intervention, in which variables such as intensity, volume, and duration can be easily manipulated. Instead, it is a behaviorally oriented and interactive approach, requiring continuous engagement with participants. Active and attentive listening played a key role in tailoring physical activity practices to individual preferences and real-life contexts. The dynamic nature of the intervention, aimed at fostering intrinsic motivation and long-term adherence—based on self-determination theory—made it challenging to define rigid protocols in advance, as the intervention was designed to be flexible and responsive to each participant’s needs and motivations. Despite these limitations, participant recruitment only began after approval from the ethics committee, and the intervention was conducted in full compliance with ethical principles, ensuring transparency and proper documentation of all study procedures.

## Supplementary Information

Supplementary Material 1.

Supplementary Material 2.

## Data Availability

Not applicable (this manuscript does not report data generation or analysis).
