# Supplementary material for: Vincular Project: study protocol for a randomized controlled trial with changes in 24-h movement behaviors targeting the reduce of depressive symptoms in adults
Source: Trials. 2026 May 15;27:474. doi: 10.1186/s13063-026-09771-8 (PMC13343858; doi:10.1186/s13063-026-09771-8)
Supplement: Supplementary file 2 — Supplementary Material 2. [file 13063_2026_9771_MOESM2_ESM.doc]

# *supplementary material*

MEETING 1 – INTRODUCTORY LESSON

Aim: to present the program schedule and meet the participants.

Content:

- Presentation: team and projects (10 minutes);

- Presentation: timetable and authorization form (30 minutes);

- Explanations: how the materials will be made available in Google Classroom (10 minutes).

Method: lecture (presential).

Psychological needs:

• Relatedness: involvement and connection.

Intervention strategies for psychological needs:

- Considering knowing the participant`s life story;

- Engaging actively in activities, including doing and playing together;

- Valuing group activities.

Class dynamics: the speaker should throw a ball to a random person, who should introduce themselves (name, age, what they do and something they think is relevant to the group). The person who has just introduced themselves throws the ball to someone else and so on (30 to 40 minutes).

Take-home dynamics:

• Answer the "Creating bonds" form (https://forms.gle/m3CdE84QFrPpB1Ns6), which is made up of 13 questions to better understand the reality of each participant, such as: the neighborhood where they live and work, phobias, the time they take off work, their occupation, family members who may be present at a meeting and whether they are available to meet at the weekend;• Dynamics of similarities: in pairs or trios, the participants should chat via Whatsapp with the aim of getting to know each other a little better and finding two similarities and two differences, which will be discussed at the next meeting.

Resources: projector, authorization form, printed timetable, ball.

Presentation:

https://www.canva.com/design/DAFYsSVllQ8/EZhj0v-ts2yzrD6fwQZpRw/edit

Reference:

GILLISON, F. B.; ROUSE, P.; STANDAGE, M.; SEBIRE, S. J.; RYAN, R. M. A meta-analysis of techniques to promote motivation for health behaviour change from a self-determination theory perspective. **Health Psychology Review**, v. 13, n. 1, p. 110-130, 2019.

MEETING 2 – DEPRESSIVE SYMPTOMS

Aim: to talk about depressive symptoms, their determinants and coping possibilities.

Content:

• Previous meeting: returning to the subject of the last meeting and opening up space for the pairs or trios to report what they thought of the dynamics and comment on the similarities and differences found (20 to 30 minutes);

• Depression and depressive symptoms: what it is, how it came about, diagnosis and symptoms, classification (10 to 15 minutes);

• Epidemiological data: presentation of data, questioning "Brazil's position in the depression ranking" (4th place) and which countries have the highest prevalence (Ukraine, Australia and Slovakia) (5 to 10 minutes);

• Recognized and effective treatments, modifiable and non-modifiable factors associated with depressive symptoms (10 to 15 minutes).

Method: lecture (presential).

Psychological needs:

• Autonomy: provision of justification;

• Autonomy: orientation with intrinsic objective;

• Competence: promotion of education.

Intervention strategies for psychological needs:

- Reflecting on reasons (internal and external) for adhering to the programs of physical activity;
- Tracing short-term goals;
- Providing educational material containing multidisciplinary information pertinent to the

physical activity and health relationship.

Class dynamics: "Breaking Sedentary Behavior". During the presentation, participants will be asked to stand in a circle to carry out the dynamic. A rope will be placed in the middle of the circle and some depressive symptoms will be mentioned, as well as common situations among those who suffer from depressive symptoms. Those who perceive the symptoms and situations mentioned should approach the rope. For each item mentioned, the speaker can open up a space for dialogue. The aim of the dynamic is to show that the participants are not alone and that it is possible to share experiences with the group (20 to 30 minutes).

Take-home dynamics: observe movement patterns of active behavior over the weekend and, if possible, do some physical activity of the participant's own choosing.

Resources: projector, rope.

Presentation:

https://www.canva.com/design/DAFZKnAlzcw/Nlge1XXEaDGanXc5dikT3A/edit

References:

Firth, J.; Solmi, M.; Wootton, R. E.; Vancampfort, D.; Schuch, F. B.; Hoare, E.; et al. A meta‐review of “lifestyle psychiatry”: the role of exercise, smoking, diet and sleep in the prevention and treatment of mental disorders. **World Psychiatry**, v. 19, n. 3, p. 360-380, 2020.

PEARCE, M.; GARCIA, L.; ABBAS, A.; STRAIN, T.; SCHUCH, F. B.; GOLUBIC, R.; et al. Association between physical activity and risk of depression: a systematic review and meta-analysis. **JAMA psychiatry**, v. 79, n. 6, p. 550-559, 2022.

ROSENBAUM, S.; MORELL, R.; ABDEL-BAKI, A.; AHMADPANAH, M.; ANILKUMAR, T. V.; BAIE, L.; et al. Assessing physical activity in people with mental illness: 23-country reliability and validity of the simple physical activity questionnaire (SIMPAQ). **BMC Psychiatry**, v. 20, n. 1, p. 1-12, 2020.

ZHAI, L.; ZHANG, Y.; ZHANG, D. Sedentary behaviour and the risk of depression: a meta-analysis. **British Journal of Sports Medicine**, v. 49, n. 11, p. 705-709, 2015.

MEETING 3 – INTRODUCTION TO PHYSICAL ACTIVITY

Aim: to explore the concept of physical activity, its benefits, domains, most practiced types and its relationship with depression.

Content:

- Previous meeting: with the participants reporting on the physical activity task they noticed and/or practiced over the weekend (15 to 20 minutes);

- Physical activity: what it is, benefits, domains, World Health Organization recommendations and the Brazilian Physical Activity Guide (15 to 20 minutes);

- Physical activity and physical exercise: the difference between them (5 minutes);

- Physical activity and its relationship with depression, suggestions for practice (20 to 30 minutes).

Method: lecture (online).

Basic Psychological Needs:

• Autonomy: language style;

• Competence: providing challenges.

Intervention strategies for basic psychological needs:

- Ensure that the discourse is not obligatory, that it does not blame the other, or that it is not unidirectional about the desire of the professional rather than the participant;

- Provide situations that culminate in gamified processes.

Class dynamics:

- "Do you practice physical activity?". Participants should vote on whether they do any physical activity (5 minutes);

- "What are the benefits of physical activity?". Through an online platform, participants should report the benefits they know about regular physical activity and these will appear in the form of a "word cloud" (5 to 15 minutes).

Take-home dynamics: observe sedentary behavior and sleep over the course of the week.

Resources: computer with internet access, Google Meet.

Presentation:

https://www.canva.com/design/DAFZRnmSWJE/YwNK3B68gDqESuWSsGFDTQ/edit

https://www.menti.com/alkwjvt2subx

References:

NAHAS, M. V. **Atividade física, saúde e qualidade de vida**: conceitos e sugestões para um estilo de vida ativo – 7. ed. – Florianópolis: Ed. do Autor, 2017.

TEYCHENNE, M.; WHITE, R. L.; RICHARDS, J.; SCHUCH, F. B.; ROSENBAUM, S.; BENNIE, J. A. Do we need physical activity guidelines for mental health: what does the evidence tell us? **Mental Health and Physical Activity**, v. 18, p. 100315, 2020.

WHITE, R. L.; PARKER, P. D.; LUBANS, D. R.; MACMILLAN, F.; OLSON, R.; ASTELL-BURT, T.; LONSDALE, C. Domain-specific physical activity and affective wellbeing among adolescents: an observational study of the moderating roles of autonomous and controlled motivation. **International Journal of Behavioral Nutrition and Physical Activity**, v. 15, n. 1, p. 87, 2018.

MEETING 4 – INTRODUCTION TO SEDENTARY BEHAVIOUR AND SLEEP

Aim: to explore the concept of sedentary behavior, its harms, domains and most common types. To address the concept of sleep, its stages and sleep hygiene techniques.

Contents:

- Previous meeting: with the participants reporting on the sedentary behavior and sleep task (15 to 20 minutes);

- Sedentary behavior: difference between physical inactivity and sedentary behavior, domains of sedentary behavior, time recommendations, related diseases (15 to 25 minutes);

- Sleep: concept, stages and quality, sleep architecture, relationship with other diseases, emphasis on depression and sleep hygiene (20 to 30 minutes).

Method: lecture (online).

Basic psychological needs:

• Autonomy: language style;

• Competence: providing challenges.

Intervention strategies for basic psychological needs:

- Ensure that the discourse is not obligatory, that it does not blame the other, or that it is not unidirectional about the desire of the professional rather than the participant;

- Providing situations that culminate in gamified processes.

Class dynamics:

- "What can we do to break sedentary behavior?". Participants should list strategies or tips on how to reduce sedentary behavior. Based on the answers obtained, this content will be published on the Vincular Project's social networks. Participants will then contribute to the creation of educational materials (5 to 10 minutes);

- "How to get a better quality of sleep?". Participants should list strategies or tips related to sleep hygiene. As with the previous activity, the answers obtained will also be used to create content for the project's social networks (5 to 10 minutes).

Take-home dynamics: think about and list general questions related to physical activity.

Resources: computer with internet access, Google Meet.

Presentation:

https://www.canva.com/design/DAFZ21FNLBQ/d9osYsSxkhmL6v773N4xyQ/edit?utm_content=DAFZ21FNLBQ&utm_campaign=designshare&utm_medium=link2&utm_source=sharebutton

References:

HALLGREN, M; NGUYEN, T.; OWEN, N.; VANCAMPFORT, D.; DUNSTAN, D. W.; WALLIN, P.; ANDERSSON, G.; EKBLOM-BAK, E. Associations of sedentary behavior in leisure and occupational contexts with symptoms of depression and anxiety. **Preventive Medicine**, v. 133, p. 106021-106027, 2020.

HALLGREN, M.; OWEN, N.; STUBBS, B.; ZEEBARI, Z.; VANCAMPFORT, D.; SCHUCH, F.; et al. Passive and mentally-active sedentary behaviors and incident major depressive disorder: a 13-year cohort study. **Journal of Affective Disorders**, v. 241, p. 579-585, 2018.

NATIONAL SLEEP FOUNDATION. **How Sleep Works:** an introduction to the key details about what happens to the mind and body during sleep. 2020. Disponível em: <https://www.sleepfoundation.org/how-sleep-works>. Acesso em: 20 de janeiro de 2022.

NATIONAL SLEEP FOUNDATION. **Stages of Sleep**. 2021. Disponível em: <https://www.sleepfoundation.org/stages-of-sleep>. Acesso em: 20 de janeiro de 2022.

SCHUCH, F. B.; VANCAMPFORT, D.; FIRTH, J.; ROSENBAUM, S.; WARD, P. B.; REICHERT, T.; et al. Physical activity and sedentary behavior in people with major depressive disorder: a systematic review and meta-analysis. **Journal of Affective Disorders**, v. 210, p. 139-150, 2017.

STUBBS, B.; VANCAMPFORT, D.; FIRTH, J.; SCHUCH, F. B.; HALLGREN, M.; SMITH, L.; et al. Relationship between sedentary behavior and depression: a mediation analysis of influential factors across the lifespan among 42,469 people in low-and middle-income countries. **Journal of Affective Disorders**, v. 229, p. 231-238, 2018.

VORVOLAKOS, T.; LEONTIDOU, E.; TSIPTSIOS, D.; MUELLER, C.; SERDARI, A.; TERZOUDI, A.; et al. The association between sleep pathology and depression: a cross-sectional study among adults in Greece. **Psychiatry Research**, v. 294, p. 113502, 2020.

ZHAI, L.; ZHANG, Y.; ZHANG, D. Sedentary behaviour and the risk of depression: a meta-analysis. **British Journal of Sports Medicine**, v. 49, n. 11, p. 705-709, 2015a.

MEETING 5 – HEALTH-RELATED PHYSICAL FITNESS

Aim: to explore the elements of health-related physical fitness, such as cardiorespiratory fitness, muscular strength/endurance, flexibility, body composition + neuromuscular fitness (balance and agility).

Content:

- Health-related physical fitness and body composition: concept, elements, applicability (15 to 20 minutes).

Method: practical class in the fitness lab and on the outdoor court (face-to-face).

Basic psychological needs:

• Autonomy: structural facilitation;

• Competence: providing encouragement and support.

Intervention strategies for basic psychological needs:

- Adjust the level of demand to the participant's ability to respond to the task;

- Adapting and constructing environmental changes that guarantee the start of the activity;

- Provide positive feedback to the class and individually, either by recognizing a participant's effort or praising their attitude.

Class dynamics:

- Warm-up: "10 passes". Two teams. One team tries to pass the ball between its members without the opposing team being able to intercept it. The aim is to make 10 consecutive passes. If the opposing team manages to catch the ball, the count is restarted and the team that intercepted it now tries to achieve the 10 passes (5 to 10 minutes);

- Cardiorespiratory fitness: in pairs, on the sides of the volleyball court, while participant "A" does jumping jacks, participant "B" takes the ball to the other side of the court and back. When they return, "B" does jumping jacks and "A" takes another ball. The idea is to run forwards and backwards. Each participant does this four times (5 to 10 minutes);

- Strength: squats with three variations, depending on the ability of the participants, which can be seated, standing or standing with dumbbells. The activity will take 2 to 3 sets of 10 to 15 repetitions, with 40 seconds rest between sets, respecting the individuality of each participant (5 minutes);

- Strength: push-ups with three variations, depending on the ability of the participants, which can be supported on the bar, with the knees bent or in the full position. The activity will take 2 to 3 sets of 10 to 15 repetitions, with 40 seconds rest between sets, respecting the individuality of each participant (5 minutes);

- Agility: "boss says so", in which participants must run a stationary race and move forwards, backwards, to one side or the other when the teacher asks them to. The aim is to make the movement in the shortest possible reaction time (5 minutes);

- Agility: in pairs, sitting down, the students must obey the teacher's commands and the first to catch the ball, which will be in the middle of the pair, wins (5 minutes);

- Dynamic balance and coordination: variations on the step, according to the teacher's instructions. Example: up up up + sideways sideways + up up up + backwards backwards (5 minutes);

- Static balance: airplane posture, leaning on one foot and lifting the other leg backwards. The student can hold onto the bar if they are unable to balance on their own. The same movement should be done with the opposite leg afterwards (5 minutes);

- Flexibility: final stretch, working with the general musculature of the body and increasing the range of movement of the joints (5 to 10 minutes).

- Notes: the activities can be adapted according to the abilities of the participants, the weather and conditions.

Take-home dynamics: for those who haven't done it yet, think about and list general doubts related to physical activity.

Resources: balls, bench, dumbbells, steps.

References:

AMERICAN COLLEGE OF SPORTS MEDICINE. **Diretrizes do ACSM para os testes de esforço e sua prescrição** – 8. ed. – Rio de Janeiro: Guanabara Koogan, 2010.

GARBER, C. E.; BLISSMER, B.; DESCHENES, M. R.; FRANKLIN, B. A.; LAMONTE, M. J.; LEE, I. M.; et al. Quantity and quality of exercise for developing and maintaining cardiorespiratory, musculoskeletal, and neuromotor fitness in apparently healthy adults: guidance for prescribing exercise. **Medicine & Science in Sports & Exercise**, v. 43, n. 7, p. 1334-1359, 2011.

MEETING 6 – BARRIERS AND FACILITATORS TO PHYSICAL ACTIVITY

Aim: to contribute information such as carrying out activities in special environmental conditions, the composition of an active practice and general care in the practice of physical activities.

Content:

- Barriers and facilitators to the practice of physical activity: what participants understand by this (10 to 15 minutes);

- Factors associated with physical activity (10 to 15 minutes);

- Classification of barriers into environmental, interpersonal and intrapersonal (10 minutes);

- Article by Rech et al. (2018): Perceived barriers to leisure-time physical activity in the Brazilian population (10 minutes);

- Models and theories that help in the adoption and maintenance of healthy behaviors: Stages of Behavior Change and Self-Determination Theory (30 to 40 minutes).

Method: lecture (online).

Basic psychological needs:

• Competence: facilitation to complete goal-related processes;

• Relatedness: social support.

Intervention strategies for basic psychological needs:

- Contribute different forms of conditioning to achieve the goals;

- Encourage the participant to invite friends and family to take part in physical activity.

Class dynamics: using the mentimeter platform, identify the main barriers to physical activity perceived by the participants and engage in a dialogue about them, seeking to explore how often the barriers are perceived and the main reasons for them (10 to 15 minutes).

Take-home dynamics: take advantage of the holiday and invite a family member, colleague or friend to do some physical activity and send a record (audio, video, photo, text) commenting on the experience.

Resources: computer with internet access, Google Meet.

Presentation:

https://www.canva.com/design/DAFalf1oyMI/XtQbFZlBf5NdtdvnqZJFzg/edit

https://www.mentimeter.com/app/presentation/alwkc6gx2zg7bo5uasejkmqfqiwdky47/kfbdxq2m8ai4/edit

References:

RECH, C. R.; CAMARGO, E. M. D.; ARAUJO, P. A. B. D.; LOCH, M. R.; REIS, R. S. Perceived barriers to leisure-time physical activity in the Brazilian population. **Revista Brasileira de Medicina do Esporte**, v. 24, n. 4, p. 303-309, 2018.

SCHUCH, F. B.; STUBBS, B. The role of exercise in preventing and treating depression. **Current Sports Medicine Reports**, v. 18, n. 8, p. 299-304, 2019.

VANCAMPFORT, D.; STUBBS, B.; SIENAERT, P.; WYCKAERT, S.; DE HERT, M.; ROSENBAUM, S.; PROBST, M. What are the factors that influence physical activity participation in individuals with depression? A review of physical activity correlates from 59 studies. **Psychiatria Danubina**, v. 27, n. 3, p. 0-224, 2015.

MEETING 7 – CURIOSITIES ABOUT PHYSICAL ACTIVITY

Aim: to propose a question and answer session on the world of physical activity.

Content: with all the questions sent in by the participants and other relevant questions on the subject chosen by the teachers, the "Cards on the table" dynamic will be created. Each card has a question or a challenge, such as performing some physical activity. The participant must choose a number and, when the card is revealed, read it aloud to everyone. If the card chosen is a question, the participants can engage in a dialogue in an attempt to answer the question together. If they don't know the answer, the teachers in charge help with the dynamic. If the chosen card is a challenge, everyone should do it together.

Method: lecture (online).

Basic psychological needs:

• Autonomy: language style;

• Competence: promoting education.

Intervention strategies for basic psychological needs:

- Opt for language that ensures choice about what to do, such as different types of exercise, how to adapt activities;

- Provide educational material containing multidisciplinary information.

Resources: computer with internet access, Google Meet.

Presentation:

https://wordwall.net/pt/resource/53196087

https://drive.google.com/file/d/1oKg2olYGvFMrxiyBaM9I6Oioynrvzv_C/view?usp=drive_link

Take-home dynamics: at the end of the meeting, all the questions and answers were forwarded to the participants so that they could fully verify the information discussed.

MEETING 8 – "AWAKEN THE INCREDIBLE IN ME", WITH LAINE VALGAS

Aim: to take part in the meeting "Awaken the incredible in me", given by Laine Valgas, a speaker on mental health and emotional intelligence and a specialist in neuroscience, positive psychology and mindfulness.

Content:

- Reflections on the importance of getting to know yourself and "your inner child";

- The relationship between childhood experiences and emotional issues faced in adulthood;

- Emotional support;

- Ways of taking control of our lives and being leaders of our own journeys;

- Motivational strategies to be our best version.

Method: lecture in the auditorium with external guest (face-to-face).

Basic psychological needs:

• Competence: providing feedback.

Intervention strategies for basic psychological needs:

- Recognizing the improvement achieved by the participant and recalling the "path" taken to achieve it.

Class dynamics:

- "He who dances his evils scatters". To make the participants feel more at ease and to break sedentary behavior, the lecturer can propose a choreography together;

- "Emotional support. You are not alone". In pairs, the participants should hold hands for a minute, looking into each other's eyes, being welcomed and welcoming, creating a connection between the two, while the speaker says phrases of reflection and emotional support.

Take-home dynamic: from now on, participants should challenge themselves daily and answer some reflection questions, such as why they are grateful for today, what they have learnt this day, what they have done for others and what they have done for themselves.

Resources: projector and speaker.

MEETING 9 – CONDUCTING THE INTERVENTION

Aim: to engage in a dialogue about the experiences of the first month.

Content:

- In the format of an informal conversation, the teachers ask questions relating to the content and dynamics of the lessons from the previous four weeks;

- Review of the content worked on;

- Providing positive feedback and reflecting on points that can be improved for the coming weeks.

Method: conversation over coffee (face-to-face).

Basic psychological needs:

• Competence: recognizing barriers;

• Relatedness: social support.

Intervention strategies for basic psychological needs:

- Recognize the stages of behavioral change for each participant;

- Encouraging group cohesion in activities and problem-solving.

Class dynamics:

- Knot dynamics. Standing, the participants form a circle and hold hands. They are asked to memorize who is on their right and left side. After this observation, the group should walk freely while a song of the teachers' choice is played. At the end of the song, the group should stop walking and everyone should stand exactly where they are. Then each participant should hold hands with the person next to them, respecting the initial position (right hand holding the colleague on their right and left hand holding the other colleague on their left) and without leaving the place where the music stopped. Once everyone is connected to the same team-mates, the teacher asks them to return to their natural position, in a circle, but without letting go of their hands, undoing the knot that was created. The aim of the game is to show that communication is essential for the activity to work and that, from now on, this same communication will remain in the next meetings.

Take-home dynamics: fill in a list of routine activities, listing all the activities they do on a daily basis, from waking up and having breakfast to going to work or out to dinner. Afterwards, the participants classify the activities as necessary, important, urgent and circumstantial, and report the need to increase, decrease, maintain or eliminate/delegate each one, in order to assess how they can better optimize their time and adopt new healthy behaviors in the short and long term.

Reference:

GILLISON, F. B.; ROUSE, P.; STANDAGE, M.; SEBIRE, S. J.; RYAN, R. M. A meta-analysis of techniques to promote motivation for health behaviour change from a self-determination theory perspective. **Health Psychology Review**, v. 13, n. 1, p. 110-130, 2019.

MEETING 10 – TOOLS FOR 24-HOUR MOVEMENT PATTERNS

Aim: to provide information on resources that help promote physical activity, reduce sedentary behavior and sleep quality.

Content:

- Review of the content covered in previous meetings on the components of 24-hour movement behavior;

- Information on how technology can be useful and favorable for health promotion, for example through apps and streams that address this issue.

- Physical activity: apps for starting and/or improving physical activity levels, such as: "Seven - 7-minute workout", "Fitness Point", "BodBot personalized workouts", "Adidas Training: HIIT Workout", "Nike Training Club - Workouts". Additional suggestion of a social network for physical activity ("Strava");

- Sedentary behavior: apps that reduce sedentary behavior, such as "Focus to-do: pomodoro & tasks", "Home Fit", as well as features to limit time on social networks and other functions available on the mobile device;

- Sleep: suggestions for guided meditation, such as "Headspace: sleep meditation", "Relax Melodies", "Calming Music" and the music streaming platform "Spotify", with specific playlists for meditation and relaxing music, and video "Netflix".

- To contextualize the content of the next meeting (exergames), provide information on how the "Just Dance Now" app works.

Method: lecture and practice (online).

Basic psychological needs:

• Autonomy: provision of choice;

• Relatedness: social support.

Intervention strategies for basic psychological needs:

- Encourage the participant to have and perceive choice in the conduct of all the stages relating to the physical activity program;

- Encourage the participant to invite friends and family to take part in the home dynamics.

Class dynamics:

- As a way of breaking sedentary behavior, a practical activity can be carried out. The teachers show a video available on YouTube of a physical activity (https://www.youtube.com/watch?v=Gs7H9oRGeiY) that all the participants can do together;

- A guided Ho'oponopono meditation at the end of the meeting. Ho'oponopono is a technique that works on inner peace, love, forgiveness, gratitude and the healing of hurts or other negative feelings. It consists of a mental and spiritual cleansing practice that can be carried out by anyone, anytime, anywhere.

Take-home dynamic: participants should choose an app of their interest that addresses one or more components of the 24-hour movement behaviors, make it available on the Vincular Project Whatsapp group so that other colleagues can also have other options and comment on their experience of using it. If possible, invite someone to practice together.

Resources: computer with internet access, Google Meet.

Presentation:

https://www.canva.com/design/DAFcRsf7Cf8/dXFzH022fzdgUvZi3j59vw/edit) https://www.youtube.com/watch?v=Gs7H9oRGeiY https://www.youtube.com/watch?v=UOHr2BUjtjs

References:

ALDENAINI, N.; OYEBODE, O.; ORJI, R.; SAMPALLI, S. Mobile phone-based persuasive technology for physical activity and sedentary behavior: a systematic review. **Frontiers in Computer Science**, v. 2, p. 19-36, 2020.

MOULAEI, K.; BAHAADINBEIGY, K.; MASHOOF, E.; DINARI, F. Design and development a mobile-based self-care application for patient with depression and anxiety disorders: an applied and developmental study. **BMC Medical Informatics and Decision Making**, v. 23, n. 1, 2022.

PRADAL-CANO, L.; LOZANO-RUIZ, C.; PEREYRA-RODRÍGUEZ, J. J.; SAIGÍ-RUBIÓ, F.; BACH-FAIG, A.; ESQUIUS, L.; et al. Using mobile applications to increase physical activity: a systematic review. **International Journal of Environmental Research and Public Health**, v. 17, n. 21, p. 8238, 2020.

RODRÍGUEZ-GONZÁLEZ, P.; IGLESIAS, D.; FERNANDEZ-RIO, J.; GAO, Z. Effectiveness of interventions using apps to improve physical activity, sedentary behavior and diet: an umbrella review. **Complementary Therapies in Clinical Practice**, v. 50, p. 101711-101717, 2022.

SILVA, A. G.; SIMOES, P.; QUEIROS, A.; ROCHA, N.; RODRIGUES, M. Effectiveness of mobile applications running on smartphones to promote physical activity: a systematic review with meta-analysis. **International Journal of Environmental Research and Public Health**, v. 17, n. 7, p. 2251, 2020.

MEETING 11 – EXERGAMES

Aim: to provide and explore a new tool for practicing physical activity, using all the elements of the corporal culture of movement.

Content:

- Brief explanation of what exergames are, costs and how to set them up;

- Practical part covering all the elements of the body culture of movement: dance, fighting, sport, gymnastics and games using the "Kinect Sports" and "Just Dance" games.

Method: practical class using the "Xbox 360" video game console (in person).

Basic psychological needs:

• Relatedness: involvement and connection.

Intervention strategies for basic psychological needs:

- Actively engaging in activities, including doing and playing together.

Resources: two projectors, two speakers and two "Xbox 360" video game consoles with the games: "Kinect Sports" and "Just Dance".

References:

CUGUSI, L.; PROSPERINI, L.; MURA, G. Exergaming for quality of life in persons living with chronic diseases: a systematic review and meta‐analysis. **PM&R**, v. 13, n. 7, p. 756-780, 2021.

HUANG, K.; ZHAO, Y.; HE, R.; ZHONG, T.; YANG, H.; CHEN, Y.; et al. Exergame-based exercise training for depressive symptoms in adults: a systematic review and meta-analysis. **Psychology of Sport and Exercise**, v.63, p. 102266-102276, 2022.

LI, J.; THENG, Y.; FOO, S. Effect of exergames on depression: a systematic review and meta-analysis. **Cyberpsychology, Behavior, and Social Networking**, v. 19, n. 1, p. 34-42, 2016.

MEETING 12 – LECTURE "SHORT, FIRM STEPS: AN INVITATION TO SELF-CARE WITH COMPASSION", WITH DUDA WERNER

Aim: to take part in the meeting "Short, firm steps: an invitation to self-care with compassion", given by Duda Werner, a graduate in physical education, coach, motivator of people, creator of the Injeção de Ânimo Project: physical and mental health.

Content:

- Reflections on dreams and talents;

- The need to identify good things or important events in day-to-day activities;

- Discussions about how many actions the participants have in their days or weeks that are dedicated exclusively to them;

- Discussions about how many of the participants' daily thoughts are to be praised;

- How to take short, firm steps from self-care with compassion in the spheres of mind, body and spirit.

Method: lecture with external guest (face-to-face).

Basic psychological needs:

• Competence: facilitation to complete relative processes and goals.

Intervention strategies for basic psychological needs:

- Centralize goals in the process;

- Valuing self-comparison as opposed to comparison with peers.

Class dynamics:

- Presentation: the participants should introduce themselves, but with a few rules. In order to value the individual as they are and what they like, they should not define themselves based on their professional life, but make a gentle and affectionate presentation, taking into account other aspects and spheres of life;

- Self-knowledge dynamics, listing qualities and things that make them happy;

- Dream list, a moment for each participant to reflect on their short-, medium- and long-term dreams.

Take-home dynamics: "happiness box". Participants should list on a piece of paper various activities, things they like to do and that make them happy, cut out each one and keep them folded in a box. When they're having a bad day, feel the need for a reward or realize that they need to celebrate some everyday achievement, they can open the box, take out a small piece of paper and do what it says.

Resources: projector, speaker, paper and colored pens.

MEETING 13 – ENVIRONMENT, PHYSICAL ACTIVITY AND THEIR POSSIBILITIES

Aim: to explore and recognize public spaces based on the characteristics related to resources, conditions, access, aesthetics and safety; to discover the diversity of physical activity practices (collective vs. individual, outdoors vs. indoors, recreational vs. competitive, with vs. without supervision).

Content:

- Reflections of the environment and explanation of what studies have found on this subject, mainly in the national context;

- Contextualization of the environment in intervention studies. Many behavioral clinical trials focus only on the individual and little has been seen about the context, way of life and interaction with the environment (built, natural, social/interaction-oriented). The combination of these three environments can create an affective-emotional environment with more meaning and intentionality;

- Considerations on the increase in life expectancy and the preparation (or not) of cities to cater for the high number of people;

- Results of a study carried out in Florianópolis, which found that 8 out of every 10 people identified in the city's parks were involved in some kind of physical activity;

- Benefits and factors associated with the use of public spaces;

- Characteristics of spaces in terms of resources, conditions, access, aesthetics, safety;

- Possibilities for practicing in public spaces and adapting activities according to certain characteristics (number of people, ability, materials, etc.).

Method: practical class in the park in the Córrego Grande neighborhood (face-to-face).

Basic psychological needs:

• Autonomy: structural facilitation and recognizing the participant's perspective;

• Competence: providing challenges.

Intervention strategies for basic psychological needs:

- Recognizing the environmental potential for exercise, ensuring multiple potentialities;

- Adapting and constructing environmental changes that guarantee the start of the activity;

- Consider participants' motives for exercising, such as preferences, expectations about health outcomes and the cognitive and affective relationships established in the context of physical activity;

- Create challenges to break the routine of the exercise sessions, whether it's to perform an exercise in a more challenging way or to carry out activities in other environments.

Class dynamics:

- Recognizing the space: observing the public space where the class is being held. At this point, participants walk around the neighborhood and observe some of the characteristics of public spaces, such as resources (equipment, programs and diversity), conditions (maintenance and incivility), access (availability and equity), aesthetics (design and attractiveness) and safety (perceived and objective). Afterwards, time is given for a discussion about what was observed and whether the participants had already had this perception of other environments at other times (15 minutes);

- Human tic-tac-toe: for this game, two or more teams need to be separated to compete against each other. Nine hula hoops are placed opposite the participants and each team is given different colored waistcoats to simulate the "X" and "O" of the traditional game. When the signal is given, the first participant in line from each team runs to the hula hoop and chooses a place to put their team's waistcoat. This participant runs back so that the next in line can go. The aim of the game is to form a sequence of three identical waistcoats (horizontally, vertically or diagonally) as quickly as possible or try to stop the other team from being able to form all three sequences. In this game, you have to think fast and act strategically in order to win (15 minutes);

- Playing with a hula hoop: all the participants should hold hands in a circle and a hula hoop should be between them. The aim of the game, which can be done recreationally (all together) or competitively (separating into different teams), is to get all the participants to pass around the hula-hoop as quickly as possible until it returns to the first person (15 minutes);

- Each participant attaches a waistcoat to the side of their trousers. The aim of the game is to steal the other participants' waistcoats, collecting as many as possible. As a variation, this activity can be carried out individually or in groups (15 minutes);

- Flag catch: the aim here is to catch the flag (which can be represented by a ball or any other object) at the end of the opposing team's field and bring it to your side of the field. However, if the participant is caught in enemy territory, they must remain motionless until another member of their team touches their hand to release them and return to the game (15 minutes).

Take-home dynamic: recognize the area in which they live by observing the place, the people who frequent it, the busiest times and what the built and perceived environment looks like.

Resources: 10 hula hoops, 20 waistcoats (10 in different colors) and a ball.

References:

FERRARI, G.; WERNECK, A. O.; DA SILVA, D. R.; KOVALSKYS, I.; GÓMEZ, G.; RIGOTTI, A.; et al. Is the perceived neighborhood built environment associated with domain-specific physical activity in Latin American adults? An eight-country observational study. **International Journal of Behavioral Nutrition and Physical Activity**, v. 17, n. 1, p. 1-14, 2020.

KOWITT, S. D.; AIELLO, A. E.; CALLAHAN, L. F.; FISHER, E. B.; GOTTFREDSON, N. C.; JORDAN, J. M.; MUESSIG, K. E. Associations among neighborhood poverty, perceived neighborhood environment, and depressed mood are mediated by physical activity, perceived individual control, and loneliness. **Health & Place**, v. 62, p. 102278-102294, 2020.

PONTIN, F. L.; JENNESON, V. L.; MORRIS, M. A.; CLARKE, G. P.; LOMAX, N. M. Objectively measuring the association between the built environment and physical activity: a systematic review and reporting framework. **International Journal of Behavioral Nutrition and Physical Activity**, v. 19, n. 1, p. 1-22, 2022.

STAPPERS, N. E. H.; VAN KANN, D. H. H.; ETTEMA, D.; DE VRIES, N. K.; KREMERS, S. P. J. The effect of infrastructural changes in the built environment on physical activity, active transportation and sedentary behavior–a systematic review. **Health & Place**, v. 53, p. 135-149, 2018.

MEETING 14 – CAPOEIRA

Aim: to learn about the history of capoeira, its institutionalization process and its main aspects, and to practice the basic elements and methodological aspects of teaching capoeira.

Content:

- Presentation of the history of capoeira, types, clothing and curiosities about this modality;

- Practical initiation exercises for individuals, pairs and groups;

- Ginga and attack and defense movements.

Method: workshop with teachers from the Capoeira Angola Palmares group (in person).

Basic psychological needs:

• Competence: providing challenges;

• Relatedness: involvement and connection.

Intervention strategies for basic psychological needs:

- Creating challenges to break the routine of exercise sessions, whether it's performing an exercise in a more challenging way or carrying out activities in other environments;

- Valuing group activities.

Resources: speaker, steps, elastic bands.

References:

AMITAY, G. Capoeira clubs as inclusive and therapeutic communities for youth and young adults experiencing social exclusion. **International Journal of Qualitative Studies in Education**, p. 1-17, 2022.

DELATTRE, B.; COLLAER, M. (Post-print) Capoeira: The Relationship of an Afro-Brazilian Cooperative Movement Art to State Anxiety, State Self-Efficacy, And Prosocial Behavior Tendencies. **Functional Neurology, Rehabilitation, and Ergonomics**, v. 7, n. 4, p. 61-65, 2023.

JORDAN, M.; WRIGHT, E. J.; PURSER, A.; GRUNDY, A.; JOYES, E.; WRIGHT, N.; et al. Capoeira for beginners: self-benefit for, and community action by, new capoeiristas. **Sport, Education and Society**, v. 24, n. 7, p. 756-769, 2018.

MARTINS, S. E.; LUIZ, M. E. T.; CASTRO FRANZONI, W. C.; MARINHO, A. Traditional capoeira street circles in Florianópolis (Southern Brazil): impacts of the covid-19 pandemic. **Leisure Studies**, v. 42, n. 3, p. 367-382, 2022.

MEETING 15 – LES MILLS

Aim: to provide more vigorous forms of physical activity, based on a series of high-intensity interval training (HIIT) exercises.

Content:

- Under the guidance of Maori gym professionals, participants will be able to try out the "Power Jump" and "Bodyattack" modalities;

- Information about the emergence of Les Mills, the main types of classes, how the professionals are trained to teach the classes, and the main components of health-related physical fitness will be explored.

Method: practice at the Maori academy (face-to-face).

Basic psychological needs:

• Autonomy: structural facilitation.

Intervention strategies for basic psychological needs:

- Adjusting the level of demand to the participant's ability to respond to the task.

Resources: all resources were provided by the Maori academy, mirrors, sound box and trampoline.

References:

CUNHA, P. G. **Afetividade de Praticantes de Ginástica Coletiva de uma Academia de Florianópolis**. Trabalho de Conclusão de Curso (graduação) – Universidade Federal de Santa Catarina, Centro de Desportos, Educação Física Bacharelado, 2023.

JONES, L.; KARAGEORGHIS, C. I.; LANE, A. M.; BISHOP, D. T. The influence of motivation and attentional style on affective, cognitive, and behavioral outcomes of an exercise class. **Scandinavian Journal of Medicine & Science in Sports**, v. 27, n. 1, p. 124-135, 2017.

ZUREIGAT, A.; FATTAH, O. A.; ELAYYAN, A. Effects of bodypump exercise on the emotions and life satisfaction among women during the coronavirus pandemic. **Sport Mont**, v. 19, n. 2, p. 89-94, 2021.

MEETING 16 – VOLLEYBALL

Aim: to propose a collective activity with a ball, recognizing and executing the main technical fundamentals of volleyball.

Content:

- History of volleyball;

- Technical fundamentals of the sport.

Method: practice in the gym at the Federal University of Santa Catarina (face-to-face).

Basic psychological needs:

• Competence: providing encouragement and support;

• Relatedness: involvement and connection.

Intervention strategies for basic psychological needs:

- Provide positive feedback to the class and individually, either by recognizing a participant's effort or praising their attitude;

- Valuing group activities.

Class dynamics:

- Playing with theory: the aim is to start a brief warm-up based on questions and answers about volleyball. The teams formed should position themselves at the end of the volleyball court. The teacher, positioned at the other end of the court, asks questions about the sport and the first participant from each team who knows the answer must run to the middle of the court (net), back to their team-mates and run to the teacher to answer. The participant who hits the teacher's hand first has the right to answer. If they get it wrong, the other participants from the opposing teams can answer.

○ How many players in a team are on the court? Answer: 6;

○ How many sets does a match have? Answer: 5;

○ How many points are there in a normal set? Answer: 25;

○ And when the game goes to the fifth set, how many points are there? Answer: 15;

○ What is the libero's job? Answer: to defend the opposing team's attack;

What are the five fundamentals of volleyball? Answer: serve, reception/defence, lift, attack and block;

What is the official size of the volleyball court? Answer: 9 x 18 metres.

- Warm-up: the participants should move along the lines of the volleyball court:

○ Running forwards along the side line (until they reach the net);

○ Moving sideways facing the net (on both sides of the court);

○ Running backwards (leaving the net and going to the back of the court along the side line);

○ Rotating the arms forwards;

○ Rotating the arms backwards.

- Touch drill: pairs, each positioned on the side lines of the court, facing each other. The first of the pair throws the ball up and touches it to their partner. The latter holds the ball and makes the same movement.

- Headlock drill: the same drill as the previous one, but with a headlock.

- Control drill: same position as the previous drill. The first player in the pair touches the ball up into the air and passes it to their partner. The latter can either hold the ball and start again or take it straight away.

- Mini courts: four columns. Dominate, pass and return to the end of the row.

- Mini game: on the mini courts, in trios, whoever enters serves and the game is only one point.

- Game: on the whole court, with six participants on each side.

Resources: volleyball net and balls.

References:

GUO, S.; LIU, F.; SHEN, J.; WEI, M.; YANG, Y. Comparative efficacy of seven exercise interventions for symptoms of depression in college students: a network of meta-analysis. **Medicine**, v. 99, n. 47, p. e23058, 2020.

MOHAMMADI, M. A study and comparison of the effect of team sports (soccer and volleyball) and individual sports (table tennis and badminton) on depression among high school students. **Australian Journal of Basic and Applied Sciences**, v. 5, n. 12, p. 1005-1011, 2011.

VACCARO, M. G.; Bertollo, M.; Guidetti, L.; Quattrone, A.; Emerenziani, G. P. Individuals’ depression and anxiety might be influenced by the level of physical activity and expertise: a pilot study on elite volleyball players and amateur athletes. **Sport Sciences for Health**, v. 17, p. 999-1005, 2021.

MEETING 17 – CONDUCTING THE INTERVENTION

Aim: to discuss the positive and negative aspects in relation to the intervention and the changes in behavior.

Content:

- In the format of an informal conversation, the teachers ask questions regarding the content and dynamics of the lessons from the previous four weeks;

- Review of the content worked on;

- Providing positive feedback and reflecting on points that can be improved for the coming weeks.

Method: conversation over coffee (face-to-face).

Basic psychological needs:

• Autonomy: emphasis on responsibility;

• Competence: providing feedback.

Intervention strategies for basic psychological needs:

- Make the participant aware of the need to move forward in the dynamics involved in the physical activity program;

- Reflect with the participants on aspects that can still be improved; visualize how to proceed/continue.

Class dynamics: praise board. Knowing that participants have difficulty identifying and recognizing their own qualities, each participant should praise their colleagues, creating a repertoire of positive characteristics for each one. The chart is then passed on to the participants.

Reference:

GILLISON, F. B.; ROUSE, P.; STANDAGE, M.; SEBIRE, S. J.; RYAN, R. M. A meta-analysis of techniques to promote motivation for health behaviour change from a self-determination theory perspective. **Health Psychology Review**, v. 13, n. 1, p. 110-130, 2019.

MEETING 18 – WEIGHT TRAINING

Aim: to propose a resistance training class, using resources available in the gym (dumbbells and machines).

Content:

- Review of the content of health-related physical fitness;

- Difference between physical activity and physical exercise;

- Difference between prescription and recommendation;

- Elements for prescription: frequency, intensity, time and type;

- Information on preliminary health assessment;

- Physical assessment;

- Practice in the gym, explaining which muscles are required in each exercise, possible variations and the reasons why the professional changes the order of the exercises on the training sheets, as well as clearing up any doubts.

Method: practical class in the gym at the Sports Centre of the Federal University of Santa Catarina (in person).

Basic psychological needs:

• Autonomy: provision of choice;

• Relatedness: involvement and connection.

Intervention strategies for basic psychological needs:

- Encourage the participant to have and perceive choice in the conduct of all the stages relating to the physical activity program;

- Actively engaging in activity, including doing and playing together.

Class dynamics:

- Joint warm-up;

- Practice in the gym (2 to 3 sets of 10 to 12 repetitions with 30 to 45 seconds rest between intervals):

○ Dumbbell bench press;

Low row;

○ Lateral raise;

Dumbbell curl;

Triceps on pulley (rope);

Leg press 45º;

Extension chair;

Flexor chair;

Adductor chair;

Abductor chair;

Calf raises.

Take-home dynamics: do 3 sets of 10 to 12 repetitions of the conventional abdominal crunch and 3 sets of 20 to 30 seconds of plank.

Resources: equipment available at the gym.

References:

BENNIE, J. A.; TEYCHENNE, M. J.; DE COCKER, K.; BIDDLE, S. J. Associations between aerobic and muscle-strengthening exercise with depressive symptom severity among 17,839 US adults. **Preventive Medicine**, v. 121, p. 121-127, 2019.

CARNEIRO, L.; AFONSO, J.; RAMIREZ-CAMPILLO, R.; MURAWSKA-CIAŁOWCIZ, E.; MARQUES, A.; CLEMENTE, F. M. The effects of exclusively resistance training-based supervised programs in people with depression: a systematic review and meta-analysis of randomized controlled trials. **International Journal of Environmental Research and Public Health**, v. 17, n. 18, p. 6715-6737, 2020.

GORDON, B. R.; MCDOWELL, C. P.; HALLGREN, M.; MEYER, J. D.; LYONS, M.; HERRING, M. P. Association of efficacy of resistance exercise training with depressive symptoms: meta-analysis and meta-regression analysis of randomized clinical trials. **Jama Psychiatry**, v. 75, n. 6, p. 566-576, 2018.

MARQUES, A.; GOMEZ-BAYA, D.; PERALTA, M.; FRASQUILHO, D.; SANTOS, T.; MARTINS, J.; et al. The effect of muscular strength on depression symptoms in adults: a systematic review and meta-analysis. **International Journal of Environmental Research and Public Health**, v. 17, n. 16, p. 5674, 2020.

MEETING 19 – FUNCTIONAL TRAINING

Aim: to offer a meeting focused on activities related to control, stability and motor coordination, contributing to the performance of habitual activities.

Content:

- Review of the content of health-related physical fitness;

- Review of the difference between prescription and recommendation;

- Review of the elements for prescription: frequency, intensity, time and type;

- Difference between resistance training (weight training) and functional training;

- Benefits of functional training;

- Practice, explaining which muscles are required in each exercise, possible variations and which day-to-day activities require the same muscles, as well as clearing up any doubts.

Method: practical class in circuit format in the fitness laboratory (in person).

Basic psychological needs:

• Autonomy: structural facilitation;

• Competence: facilitation to complete goal-related processes.

Intervention strategies for basic psychological needs:

- Recognizing the environmental potential for exercise, ensuring multiple potentialities;

- Create different forms of conditioning to achieve goals.

Class dynamics:

- Joint warm-up:

○ Ankle 10x each side;

○ Hip 10x each side;

○ Knee raised upwards 3x on each side;

○ Knee raised backwards 3x on each side;

○ Jumping jack 3x of 10 to 12 repetitions;

○ Arm rotation forwards;

○ Arm rotation backwards;

○ Trunk rotation;

Progressive running: going forwards and returning backwards.

- Practice in a circuit format (30 seconds of running and 30 seconds to change stations):

○ Climber with handstand on bench + jump on bench;

○ Abdominal rowing;

○ Support / flexion on the bar used in dance classes;

○ Pull-up / inverted crucifix;

○ Dips + lateral raises;

○ With a step and two dumbbells: extend your legs backwards (keeping your fists on the set), do the sawing movement with the dumbbells (interleaving the upper limbs), stand on the step and rise to the vertical position;

○ Plyometrics, jumping with both feet over the boundary;

○ Abdominal plank;

○ Coordination plus squat: with two hula hoops side by side on the floor, the participant places their left leg on the left hula hoop, their right leg on the right hula hoop, returns to the starting position with the left, returns with the right, performs the same movement again and then does a squat;

○ Juggling: with two tennis balls in each hand, the participant must throw them up in the air and change hands;

○ Jumping rope.

Resources: ropes, elastic bands, balls, mats, benches, steps, dumbbells.

References:

AMERICAN COLLEGE OF SPORTS MEDICINE. **Diretrizes do ACSM para os testes de esforço e sua prescrição** – 8. ed. – Rio de Janeiro: Guanabara Koogan, 2010.

GARBER, C. E.; BLISSMER, B.; DESCHENES, M. R.; FRANKLIN, B. A.; LAMONTE, M. J.; LEE, I. M.; et al. Quantity and quality of exercise for developing and maintaining cardiorespiratory, musculoskeletal, and neuromotor fitness in apparently healthy adults: guidance for prescribing exercise. **Medicine & Science in Sports & Exercise**, v. 43, n. 7, p.

1334-1359, 2011.

MEETING 20 – DANCE

Aim: to experience different dance practices.

Content:

- Dance initiation, with the modalities of samba no pé, samba de gafieira and Zouk.

Method: workshop with a guest dance teacher (in person).

Basic psychological needs:

• Competence: providing challenges.

Intervention strategies for basic psychological needs:

- Creating challenges to break up the routine of the exercise sessions, either to perform an exercise in a more challenging way or to carry out activities in other environments.

Resources: sound box.

References:

HELLEM, T.; SUNG, Y. H.; FERGUSON, H.; HILDRETH, L. The emotional dance with depression: a longitudinal investigation of OULA® for depression in women. **Journal of Bodywork and Movement Therapies**, v. 24, n. 4, p. 413-422, 2020.

KARKOU, V.; AITHAL, S.; ZUBALA, A.; MEEKUMS, B. Effectiveness of dance movement therapy in the treatment of adults with depression: a systematic review with meta-analyses. **Frontiers in Psychology**, v. 10, p. 936-959, 2019.

MEETING 21 – YOGA

Aim: to try out the basic yoga positions and do a relaxation practice.

Content:

- History and benefits of yoga;

- Initiation practice and breathing exercises.

Method: workshop with a guest yoga teacher (in person).

Basic psychological needs:

• Autonomy: structural facilitation;

• Competence: providing encouragement and support.

Intervention strategies for basic psychological needs:

- Adjust the level of demand to the participant's ability to respond to the task;

- Provide positive feedback to the class and individually, either by recognizing a participant's effort or praising their attitude.

Resources: speaker, mats or yoga mats.

References:

BREEDVELT, J. J.; AMANVERMEZ, Y.; HARRER, M.; KARYOTAKI, E.; GILBODY, S.; BOCKTING, C. L.; et al. The effects of meditation, yoga, and mindfulness on depression, anxiety, and stress in tertiary education students: a meta-analysis. **Frontiers in Psychiatry**, v. 10, p. 193-208, 2019.

BRINSLEY, J.; SCHUCH, F.; LEDERMAN, O.; GIRARD, D.; SMOUT, M.; IMMINK, M. A.; et al. Effects of yoga on depressive symptoms in people with mental disorders: a systematic review and meta-analysis. **British Journal of Sports Medicine**, v. 55, n. 17, p. 992-1000, 2021.

CRAMER, H.; ANHEYER, D.; LAUCHE, R.; DOBOS, G. A systematic review of yoga for major depressive disorder. **Journal of Affective Disorders**, v. 213, p. 70-77, 2017.

NANTHAKUMAR, C. Yoga for anxiety and depression: a literature review. **The Journal of Mental Health Training, Education and Practice**, v. 15, n. 3, p. 157-169, 2020.

MEETING 22 – SLACKLINE

Aim: to work with different forms of balance from the practice of slackline.

Content:

- Introduction to slackline;

- Importance/benefits of carrying out physical activities outdoors;

- Information on adventure activities;

- Publicizing the extension project.

Method: workshop with those responsible for the extension project at the Federal University of Santa Catarina (face-to-face).

Basic psychological needs:

• Autonomy: orientation with an intrinsic goal.

Intervention strategies for basic psychological needs:

- Rationalizing the role of physical activity for reasons such as building a sense of friendship, improving skills, gaining energy and a better lifestyle.

Class dynamics:

- Stretching exercises, individually and in pairs;

- Balance exercises with bosu and balance platform;

- Slackline practice, with individual exercises, in pairs, in trios and with all participants involved.

Resources: sound box, bosu, slackline.

References:

BRITO, H. S.; CARRACA, E. V.; PALMEIRA, A. L.; FERREIRA, J. P.; VLECK, V.; ARAUJO, D. Benefits to performance and well-being of nature-based exercise: a critical systematic review and meta-analysis. **Environmental Science & Technology**, v. 56, n. 1, p. 62-77, 2021.

COVENTRY, P. A.; BROWN, J. E.; PERVIN, J.; BRABYN, S.; PATEMAN, R.; BREEDVELT, J.; et al. Nature-based outdoor activities for mental and physical health: systematic review and meta-analysis. **SSM-Population Health**, v. 16, p. 100934, 2021.

FRÜHAUF, A.; NIEDERMEIER, M.; ELLIOTT, L. R.; LEDOCHOWSKI, L.; MARKSTEINER, J.; KOPP, M. Acute effects of outdoor physical activity on affect and psychological well-being in depressed patients–a preliminary study. **Mental Health and Physical Activity**, v. 10, p. 4-9, 2016.

MEETING 23 –CYCLING TOUR

Aim: to carry out a group practice in the open air.

Content:

- Importance/benefits of carrying out physical activities in groups and outdoors.

Method: practical activity in the city of Florianópolis/SC (in person).

Basic psychological needs:

• Relatedness: involvement and connection.

Intervention strategies for basic psychological needs:

- Valuing group activities.

Class dynamics: together with the group, a cycle ride was organized to and from the Federal University of Santa Catarina to the northern seafront of Florianópolis/SC.

Resources: bicycles, safety equipment for each participant, terms of responsibility.

References:

BRITO, H. S.; CARRACA, E. V.; PALMEIRA, A. L.; FERREIRA, J. P.; VLECK, V.; ARAUJO, D. Benefits to performance and well-being of nature-based exercise: a critical systematic review and meta-analysis. **Environmental Science & Technology**, v. 56, n. 1, p. 62-77, 2021.

COVENTRY, P. A.; BROWN, J. E.; PERVIN, J.; BRABYN, S.; PATEMAN, R.; BREEDVELT, J.; et al. Nature-based outdoor activities for mental and physical health: systematic review and meta-analysis. **SSM-Population Health**, v. 16, p. 100934, 2021.

FRÜHAUF, A.; NIEDERMEIER, M.; ELLIOTT, L. R.; LEDOCHOWSKI, L.; MARKSTEINER, J.; KOPP, M. Acute effects of outdoor physical activity on affect and psychological well-being in depressed patients–a preliminary study. **Mental Health and Physical Activity**, v. 10, p. 4-9, 2016.

MATIAS, T. S.; LOPES, M. V. V.; COSTA, B. G.; SILVA, K. S.; SCHUCH, F. B. Relationship between types of physical activity and depression among 88,522 adults. **Journal of Affective Disorders**, v. 297, p. 415-420, 2022.

MEETING 24 – SAND SPORTS

Aim: to provide an encounter in which participants have the opportunity to try the sport of beach tennis.

Content:

- Explanation of the sport;

- Presentation of equipment;

- Teaching the main fundamentals.

Method: practical activity at Arena Beach Floripa with professionals in the field (in person).

Basic psychological needs:

• Autonomy: structural facilitation;

• Relatedness: co-operation group.

Intervention strategies for basic psychological needs:

- Adjusting the level of demand to the participant's ability to respond to the task;

- Creating a group to exchange experiences, facilitating communication between participants.

Class dynamics:

- Warm-up;

- Ball handling exercises;

- Serving exercises;

- Moving exercises;

- Mini game.

Resources: beach tennis rackets, net and balls, all provided by Arena Beach Floripa.

References:

BRITO, H. S.; CARRACA, E. V.; PALMEIRA, A. L.; FERREIRA, J. P.; VLECK, V.; ARAUJO, D. Benefits to performance and well-being of nature-based exercise: a critical systematic review and meta-analysis. **Environmental Science & Technology**, v. 56, n. 1, p. 62-77, 2021.

COVENTRY, P. A.; BROWN, J. E.; PERVIN, J.; BRABYN, S.; PATEMAN, R.; BREEDVELT, J.; et al. Nature-based outdoor activities for mental and physical health: systematic review and meta-analysis. **SSM-Population Health**, v. 16, p. 100934, 2021.

FRÜHAUF, A.; NIEDERMEIER, M.; ELLIOTT, L. R.; LEDOCHOWSKI, L.; MARKSTEINER, J.; KOPP, M. Acute effects of outdoor physical activity on affect and psychological well-being in depressed patients–a preliminary study. **Mental Health and Physical Activity**, v. 10, p. 4-9, 2016.

MEETING 25 – CONDUCTING THE INTERVENTION

Aim: to discuss the positive and negative aspects in relation to the intervention.

Content:

- In the format of an informal conversation, the teachers ask questions relating to the content and dynamics of the lessons from the previous four weeks;

- Review of the content worked on;

- Providing positive feedback and reflecting on points that can be improved for the coming weeks.

Method: conversation over coffee (face-to-face).

Basic psychological needs:

• Autonomy: providing justification; guidance with an intrinsic goal;

• Competence: providing feedback.

Intervention strategies for basic psychological needs:

- Evaluate and re-evaluate long-term goals;

- Recognize internal motives related to physical activity, such as satisfaction and pleasure;

- Recognizing the improvement achieved by the participant and recalling the "path" taken to achieve it.

Dynamics in the room:

- As a form of social interaction, messages are projected in video format, recorded by the participants' family members, telling a little more about the participant and their perceptions of Vincular Project in the participant's life.

References:

GILLISON, F. B.; ROUSE, P.; STANDAGE, M.; SEBIRE, S. J.; RYAN, R. M. A meta-analysis of techniques to promote motivation for health behaviour change from a self-determination theory perspective. **Health Psychology Review**, v. 13, n. 1, p. 110-130, 2019.

MEETING 26 – PET DAY

Aim: to dialogue about the relationship between animals and people living with depressive symptoms.

Content:

- Mental health benefits of having contact with pets;

- Active practices that can be carried out in the presence of pets;

- Studies involving this theme.

Method: practical activity by the Federal University of Santa Catarina (face-to-face).

Basic psychological needs:

• Relatedness: social support; involvement and connection.

Intervention strategies for basic psychological needs:

- Encouraging participants to invite friends and family to take part in physical activity programs;

- Valuing group activities.

In-class dynamics: walk with the participants' pets around the Federal University of Santa Catarina.

References:

BROOKS, H. L.; RUSHTON, K.; LOVELL, K.; BEE, P.; WALKER, L.; GRANT, L.; ROGERS, A. The power of support from companion animals for people living with mental health problems: a systematic review and narrative synthesis of the evidence. **BMC Psychiatry**, v. 18, n. 1, p. 1-12, 2018.

FRIEDMAN, E.; KRAUSE-PARELLO, C. A. Companion animals and human health: benefits, challenges, and the road ahead for human-animal interaction. **Revue Scientifique et Technique (International Office of Epizootics)**, v. 37, n. 1, p. 71-82, 2018.

KAMIOKA, H.; OKADA, S.; TSUTANI, K.; PARK, H.; OKUIZUMI, H.; HANDA, S.; et al. Effectiveness of animal-assisted therapy: a systematic review of randomized controlled trials. **Complementary Therapies in Medicine**, v. 22, n. 2, p. 371-390, 2014.

MEETING 27 – QUALITY OF LIFE FOR WORKERS AND ACTIVE AGEING

Aim: to cover content such as the assessment of the worker's quality of life, the profile of the work environment and considerations and the importance of leisure, as well as content on guidance for an active lifestyle in old age maintaining good habits for life.

Contents:

- Work, leisure and health;

- Active leisure;

- Active ageing;

- The difference between chronological and biological age;

- Factors that threaten the well-being of the elderly;

- Determinants of quality ageing;

- Benefits of physical activity for healthy ageing, physiological, psychological and social benefits;

- Guidelines for an active lifestyle in old age;

- Good habits throughout life.

Method: lecture with a guest lecturer from the Sports Centre of the Federal University of Santa Catarina (online).

Basic psychological needs:

• Autonomy: recognizing the participant's perspective.

Intervention strategies for basic psychological needs:

- Consider participants' motives for exercising, such as preferences, expectations about health outcomes and the cognitive and affective relationships established in the context of physical activity.

Class dynamics: conversation circle.

Take-home dynamic: fill in the "Well-being Pentacle" and the "Work Environment and Conditions Profile" tools.

Resources: computer with internet access, Google Meet.

Presentation: https://www.canva.com/design/DAFh48EpICU/iGHFuDzBmH_s8s6aFGR7Fg/edit?utm_content=DAFh48EpICU&utm_campaign=designshare&utm_medium=link2&utm_source=sharebutton

References:

NAHAS, M. V. **Atividade física, saúde e qualidade de vida**: conceitos e sugestões para um estilo de vida ativo – 7. ed. – Florianópolis: Ed. do Autor, 2017.

MEETING 28 – LABOR GYMNASTICS

Aim: to talk about the concept and benefits of labor gymnastics and to carry out different practices.

Content:

- Contextualization of occupational gymnastics;

- Benefits;

- Relationship with depressive symptoms.

Method: practical activity with a professional from SEBRAE - Brazilian Micro and Small Business Support Service (in person).

Basic psychological needs:

• Autonomy: language style.

Intervention strategies for basic psychological needs:

- Opt for language that ensures choice about what to do, such as different types of exercise, how to adapt activities.

Class dynamics:

- Individual stretching;

- Group dynamics:

○ Pass with the balls;

Practicing with the stick;

Group massage.

- Relaxation.

Resources: computer, projector, speaker and materials provided by SEBRAE.

References:

CONN, V. S.; HAFDAHL, A. R.; COOPER, P. S.; BROWN, L. M.; LUSK, S. L. Meta-analysis of workplace physical activity interventions. **American Journal of Preventive Medicine**, v. 37, n. 4, p. 330-339, 2009.

LAUX, R. C.; TABELA, B. A. F.; ANTONIO, D. S.; ZANINI, D. Effect of a session of the workplace physical activity program on mood. **International Physical Medicine & Rehabilitation Journal**, v. 5, n. 4, p. 141-145, 2020.

SERRA, M. V. G. B.; PIMENTA, L. C.; QUEMELO, P. R. V. Efeitos da ginástica laboral na saúde do trabalhador: uma revisão da literatura. **Revista Pesquisa em Fisioterapia**, v. 4, n. 3, 2014.

MEETING 29 – NUTRITION AND STRESS

Aim: to provide moments of reflection and awareness with the talk "Quality of life and eating behavior: learning to deal with emotional eating", with nutritionist Gabriela Cunha.

Content:

- Quality of life and eating behavior: learning to deal with emotional eating;

- Science of nutrition and diet culture.

Method: lecture with external guest from the Federal University of Pelotas (online).

Basic psychological needs:

• Autonomy: provision of choice;

• Competence: promoting education.

Intervention strategies for basic psychological needs:

- Encouraging the participant to have and perceive choice in the conduct of all the stages relating to the behavior change process;

- Provision of educational material containing multidisciplinary information pertinent to the relationship between physical activity and health.

Class dynamics: round table discussion.

Take-home dynamics: think of collective activities for the gymkhana, which will be held at meeting 31.

Resources: computer with internet access, Google Meet.

References:

EKINCI, G. N.; SANLIER, N. The relationship between nutrition and depression in the life process: a mini-review. **Experimental Gerontology**, v. 172, p. 112072-112079, 2023.

KRIS-ETHERTON, P. M.; PETERSEN, K. S.; HIBBELN, J. R.; HURLEY, D.; KOLICK, V.; PEOPLES, S.; et al. Nutrition and behavioral health disorders: depression and anxiety. **Nutrition Reviews**, v. 79, n. 3, p. 247-260, 2021.

SINGH, O.; NAAGAR, M.; MAITY, M. K.; SHARMA, S. Role of nutrition in depression and other mental illnesses. **International Journal of Science and Research Archive**, v. 7, n. 2, p. 061-068, 2022.

MEETING 30 – GYMNASTICS FOR ALL

Aim: to propose gymnastic activities and ones that contemplate the various types of bodily manifestations, such as dance and folkloric expressions.

Content:

- Concept of gymnastics for all;

- Benefits of practice;

- Publicizing the extension project;

- Practical class with finalized choreography.

Method: practical activity with the GPT On Project fellows from the Federal University of Santa Catarina (in person).

Basic psychological needs:

• Autonomy: structural facilitation;

• Competence: providing encouragement and support.

Intervention strategies for basic psychological needs:

- Adjust the level of demand to the participant's ability to respond to the task;

- Provide positive feedback to the class and individually, either by recognizing a participant's effort or praising their attitude.

Resources: balls, hula hoops and speaker.

References:

BENTO-SOARES, D.; SCHIAVON, L. M. Gymnastics for all: different cultures, different perspectives. **Science of Gymnastics Journal**, v. 12, n. 1, p. 5-18, 2020.

MENEGALDO, F. R.; BORTOLETO, M. A. C; MATEU, M. The artistic-expressive dimension of gymnastics for all. **Science of Gymnastics Journal**, v. 15, n. 2, p. 257-268, 2023.

MENEGALDO, F. R.; BORTOLETO, M. A. C. The role of time and experience to the gymnastics for all practice: building a sense of collectivity. **Science of Gymnastics Journal**, v. 12, n. 1, p. 19-26, 2020.

MEETING 31 – GYMKHANA

Aim: to explore collective and competitive activities in an open environment.

Content: carrying out activities and games with family and friends, who are divided into teams.

Method: practical activity in the Horto Florestal in the Córrego Grande neighborhood (face-to-face).

Basic psychological needs:

• Competence: facilitation to complete goal-related processes;

• Relatedness: social support.

Intervention strategies for basic psychological needs:

- Different forms of conditioning to achieve the goal;

- Create situations that essentially culminate in the participation of family and friends in activities;

- Encouraging group cohesion in activities and problem-solving.

Class dynamics:

- Equal numbers: the two teams should sit facing each other and in the center is a ball or any other object. Each participant is given a number and these numbers are repeated to the opposing team. When the teacher calls out the number, the participants from each team must try to pick up the ball and return to their place without being caught by their opponent. The game is repeated until everyone has participated more than once and the wins are totaled.

- Three-legged race: the participants split up into trios and the participant in the middle must tie the ribbon around their ankle, joining a colleague's right leg and the other's right leg. The trio must stand firm and when the teacher gives the start signal, the players run off trying to cross the finish line.

- Egg on spoon race + balloon popping: participants from the same team line up and compete with their opponents, who play together at the same time. The aim is to cross from one line to another holding the spoon with the egg (or a ping-pong ball) in your mouth. If the ping-pong ball falls, the participant can pick it up again and restart the game, starting from the place where the ball fell. As soon as they reach the finish line, the participants must pick up a full balloon that will be available, return to the starting point and pop it with the next pair, who can then start the game. The team that completes the entire journey in the shortest time wins.

- Unscrambling the letters: anagramming is a game in which participants recombine letters from a word to form new words. Participants are given pieces of paper with the same letters and must form the word that the teacher says before the opposing team.

- Emperor penguin + target practice: participants from the same team line up and compete with their opponents, who play together at the same time. The aim is to cross from one line to another holding the ball between your legs without letting it fall, resembling a penguin. Once they reach the finish line, the participants must try to throw the ball that is with the teacher at the specified target and return to the starting point so that the next colleague can play. Scores may vary.

- Bridge with hula hoops: each team lines up with their mates inside the hula hoops on the floor. With one more hula-hoop than the number of members, the activity consists of building a bridge and overcoming the established distance without the participants falling off the hula-hoops.

- Human tic-tac-toe with balance: two teams competing together. Nine hula hoops are placed opposite the participants and each team is given different colored waistcoats to simulate the "X" and "O" of the traditional game. At the signal, the first participant in line from each team spins round 7 times with their head resting on the broomstick, picks up their waistcoat and runs to the hula hoop, choosing a place to put their team's waistcoat. This participant runs back so that the next in line can go. The aim of the game is to form a sequence of three identical waistcoats (horizontally, vertically or diagonally) as quickly as possible or try to stop the other team from being able to form all three sequences. In this game, you have to think fast and act strategically in order to win

- Bullet in the flour: one participant from each team volunteers to take part in the game, which consists of finding the bullets in the flour pot with their mouth before the opposing team does.

References:

BRITO, H. S.; CARRACA, E. V.; PALMEIRA, A. L.; FERREIRA, J. P.; VLECK, V.; ARAUJO, D. Benefits to performance and well-being of nature-based exercise: a critical systematic review and meta-analysis. **Environmental Science & Technology**, v. 56, n. 1, p. 62-77, 2021.

COVENTRY, P. A.; BROWN, J. E.; PERVIN, J.; BRABYN, S.; PATEMAN, R.; BREEDVELT, J.; et al. Nature-based outdoor activities for mental and physical health: systematic review and meta-analysis. **SSM-Population Health**, v. 16, p. 100934, 2021.

FRÜHAUF, A.; NIEDERMEIER, M.; ELLIOTT, L. R.; LEDOCHOWSKI, L.; MARKSTEINER, J.; KOPP, M. Acute effects of outdoor physical activity on affect and psychological well-being in depressed patients–a preliminary study. **Mental Health and Physical Activity**, v. 10, p. 4-9, 2016.

MEETING 32 – CLOSING TRACK

Aim: to provide moments of reflection on the trajectory of the intervention and future perspectives.

Content:

- Carrying out the Costa da Lagoa trail with family and friends;

- Fraternization lunch;

- Closing of the intervention.

Basic psychological needs:

• Autonomy: guidance with an intrinsic goal;

• Competence: providing inventiveness and support;

• Relatedness: co-operation group.

Intervention strategies for basic psychological needs:

- Rationalize the role of physical activity for reasons such as building a sense of friendship, improving skills, gaining energy and a better lifestyle;

- Provide positive feedback to the class and individually, either by recognizing a participant's effort or praising their attitude;

- Create groups in virtual environments to share experiences.

Take-home dynamic: send in a statement about your journey through the project, with your perceptions of the issues worked on during the intervention.

References:

BRITO, H. S.; CARRACA, E. V.; PALMEIRA, A. L.; FERREIRA, J. P.; VLECK, V.; ARAUJO, D. Benefits to performance and well-being of nature-based exercise: a critical systematic review and meta-analysis. **Environmental Science & Technology**, v. 56, n. 1, p. 62-77, 2021.

COVENTRY, P. A.; BROWN, J. E.; PERVIN, J.; BRABYN, S.; PATEMAN, R.; BREEDVELT, J.; et al. Nature-based outdoor activities for mental and physical health: systematic review and meta-analysis. **SSM-Population Health**, v. 16, p. 100934, 2021.
